# Comparison of the diagnostic performance of twelve noninvasive scores of metabolic dysfunction-associated fatty liver disease

**DOI:** 10.1186/s12944-023-01902-3

**Published:** 2023-09-06

**Authors:** Haoxuan Zou, Xiaopu Ma, Fan Zhang, Yan Xie

**Affiliations:** 1 Department of Gastroenterology, West China Hospital, Sichuan University, No. 37 Guoxue Alley, Chengdu, 610041 Sichuan China; 2 Health Management Center, West China Hospital, General Practice Medical Center, Sichuan University, No. 37 Guoxue Alley, Chengdu, 610041 Sichuan China

**Keywords:** Metabolic dysfunction-associated fatty liver disease, External validation of prediction models, Receiver operating characteristic curve, Net reclassification index, Integrated discrimination improvement, Decision curve analysis

## Abstract

**Background:**

The absence of distinct symptoms in the majority of individuals with metabolic dysfunction-associated fatty liver disease (MAFLD) poses challenges in identifying those at high risk, so we need simple, efficient and cost-effective noninvasive scores to aid healthcare professionals in patient identification. While most noninvasive scores were developed for the diagnosis of nonalcoholic fatty liver disease (NAFLD), consequently, the objective of this study was to systematically assess the diagnostic ability of 12 noninvasive scores (METS-IR/TyG/TyG-WC/TyG-BMI/TyG-WtHR/VAI/HSI/FLI/ZJU/FSI/K-NAFLD) for MAFLD.

**Methods:**

The study recruited eligible participants from two sources: the National Health and Nutrition Examination Survey (NHANES) 2017-2020.3 cycle and the database of the West China Hospital Health Management Center. The performance of the model was assessed using various metrics, including area under the receiver operating characteristic curve (AUC), net reclassification index (NRI), integrated discrimination improvement (IDI), decision curve analysis (DCA), and subgroup analysis.

**Results:**

A total of 7398 participants from the NHANES cohort and 4880 patients from the Western China cohort were included. TyG-WC had the best predictive power for MAFLD risk in the NHANES cohort (AUC 0.863, 95% CI 0.855–0.871), while TyG-BMI had the best predictive ability in the Western China cohort (AUC 0.903, 95% CI 0.895–0.911), outperforming other models, and in terms of IDI, NRI, DCA, and subgroup analysis combined, TyG-WC remained superior in the NAHANES cohort and TyG-BMI in the Western China cohort.

**Conclusions:**

TyG-BMI demonstrated satisfactory diagnostic efficacy in identifying individuals at a heightened risk of MAFLD in Western China. Conversely, TyG-WC exhibited the best diagnostic performance for MAFLD risk recognition in the United States population. These findings suggest the necessity of selecting the most suitable predictive models based on regional and ethnic variations.

**Supplementary Information:**

The online version contains supplementary material available at 10.1186/s12944-023-01902-3.

## Introduction

Nonalcoholic fatty liver disease (NAFLD) has emerged as the predominant etiology of chronic liver disease on a global scale, affecting approximately one-third of the world’s population [1]. Additionally, NAFLD is intricately linked with comorbidities such as diabetes, hypertension, insulin resistance (IR), dyslipidemia, and heightened susceptibility to cardiovascular disease [2–4]. As research and comprehension of NAFLD have advanced, the initial “exclusionary” concept and diagnostic criteria are no longer deemed suitable for directing clinical practice and scientific investigation. Consequently, the international hepatology community has recommended the adoption of a new disease name, metabolic dysfunction-associated fatty liver disease (MAFLD), and corresponding diagnostic criteria to supersede the original NAFLD disease name and diagnostic criteria [5, 6]. Nevertheless, the majority of MAFLD patients remain asymptomatic, underscoring the necessity to explore an efficacious tool for predicting and diagnosing fatty liver at an early stage. Noninvasive diagnostic scoring has garnered significant clinical attention in recent years, owing to its noninvasive nature, ease of use, reproducibility, and minimal operator skill requirements, which is particularly useful for early screening and assessment of MAFLD [7].

IR has been identified as a crucial factor in the pathogenesis of fatty liver disease [8, 9]. The triglyceride glucose (TyG) index, which comprises fasting plasma glucose (FPG) and triglycerides (TG), has emerged as a dependable alternative marker of IR [10, 11]. Furthermore, numerous investigations have demonstrated that TyG-related indices derived from TyG (TyG-BMI/TyG-WC/TyG-WtHR) exhibit superior predictive capability for IR, owing to the robust correlation between IR and obesity, waist circumference (WC), and waist-to-height ratio (WtHR) [12, 13]. Consequently, subsequent investigations have evaluated the diagnostic potential of TyG-related indices for NAFLD in light of these discoveries, but there were significant differences in diagnostic efficacy between articles, and the metrics assessed by these studies stopped at comparing the area under the receiver operating characteristic curve (AUC) [14–19]. Similarly, the metabolic score for insulin resistance (METS-IR) [20], a recently proposed alternative index to IR, has also been shown to have good predictive value for NAFLD [21, 22]. The predictive value of TyG-related indices and METS-IR in MAFLD should be further validated in all aspects.

Additionally, there are previously constructed noninvasive diagnostic models for NAFLD that should also be validated in MAFLD. The hepatic steatosis index (HSI) [23], developed by Lee et al. in Korea in 2010, utilizes ultrasonography and incorporates the ALT/AST ratio, BMI, and presence of diabetes as its components. Similarly, the visceral adiposity index (VAI) [24], developed in Italy in 2010 by Amato et al., employs ultrasonography as a diagnostic criterion for fatty liver and includes WC, BMI, TG, and HDL. The lipid accumulation product (LAP) [25], introduced in 2005, is an index that utilizes the National Health and Nutrition Examination Survey (NHANES) III data and comprises WC and TG as its components. The fatty liver index (FLI) is a widely utilized diagnostic model for fatty liver disease that was established in Italy in 2006 by Bedogni et al. [26]. This model employs ultrasonography as a diagnostic criterion and incorporates variables such as body mass index (BMI), waist circumference (WC), triglycerides (TG), and γ-glutamyl transferase (GGT). In 2015, the Zhejiang University index (ZJU) [27] was developed in China, which includes BMI, FPG, TG, and the ALT/AST ratio. Additionally, the Framingham steatosis index (FSI) [28] was constructed by Long et al. in 2016 in the United States based on computed tomography (CT) and includes variables such as age, sex, BMI, ALT/AST ratio, presence of hypertension, and diabetes. Jeong et al. [29] employed a sample size of 3,634 individuals from the Korean National Health and Nutrition Examination Survey (KNHANES) conducted between 2008 and 2010. They developed the KNHANES NAFLD (K-NAFLD) score, incorporating variables such as sex, WC, systolic blood pressure (SBP), FPG, TG, and ALT.

However, the majority of the noninvasive indices and models mentioned above were developed for the diagnosis of NAFLD, and their applicability to MAFLD requires further validation. Consequently, this study aims to systematically validate the diagnostic accuracy of 12 noninvasive scores for MAFLD, utilizing the NHANES dataset and the dataset from the Health Management Center of West China Hospital at Sichuan University. Upon reviewing the pertinent literature, we discovered that this study represents the most extensive investigation of noninvasive models, providing a more comprehensive evaluation.

## Materials and methods

### Data sources

The present study sourced its data from NHANES 2017-2020.3, a research initiative that employs a sophisticated, multistage, probability sampling technique to gather a representative sample for evaluating the health and nutritional status of both adults and children in the United States. The NHANES study protocol was backed by the National Center for Health Statistics (NCHS). Moreover, an additional Western China cohort was sourced from the Health Management Center of West China Hospital at Sichuan University. The study protocol was approved by the Ethics Committee of West China Hospital at Sichuan University and was conducted in accordance with the ethical principles delineated in the Declaration of Helsinki. Furthermore, the present investigation adhered to the identical methodology as delineated in the Transparent Reporting of a Multivariable Predictive Model for Individual Prognosis or Diagnosis (TRIPOD) guidelines [30].

### Laboratory measurement and clinical data

The NHANES dataset and the Health Management Center dataset of West China Hospital at Sichuan University were utilized as the primary sources for all variables, encompassing demographic parameters, anthropometric parameters, comorbidities, and laboratory factors, as expounded upon in the Supplementary information. The online supplement provides definitions of demographics, lifestyle, and comorbidities, including racial status, diabetes [31], and hypertension [32]. Formulas for calculating METS-IR [20], TyG [11], TyG-BMI [13], TyG-WC [33], TyG-WtHR [12], HSI [23], VAI [24], FLI [26], LAP [25], ZJU [27], FSI [28], and K-NAFLD [29] are shown in the online supplement as well.

### Definition of MAFLD

The utilization of the controlled attenuation parameter (CAP) via VCTE proves advantageous in identifying individuals afflicted with hepatic steatosis [34, 35]. The current investigation incorporates a threshold of CAP ≥ 258 dB/m to indicate the presence of substantial hepatic steatosis, which is based on prior research [36]. The most recent expert consensus delineates the diagnosis of MAFLD as the presence of hepatic steatosis in conjunction with one or more of the following: overweight/obesity, diabetes, or metabolic dysfunction (details are available in the Supplementary Materials) [5, 6].

### Statistical analyses

R (version 4.2.2) was used for statistical analyses. Statistical significance was defined as *P* < 0.05. Continuous variables are shown as the mean ± standard deviation (SD) and were compared by Student’s t test or the Mann‒Whitney U test. Categorical values are shown as % and were compared using the χ^2^ test.

To evaluate the predictive value of noninvasive indices and models, the investigation generated receiver operating characteristic (ROC) curves and contrasted specific parameters, such as AUC, sensitivity (SEN), specificity (SPE), positive predictive value (PPV), and negative predictive value (NPV). The Delong approach was employed to ascertain whether there were statistically significant disparities in AUC between noninvasive scores [37]. Furthermore, the present study determined optimal cutoff values utilizing the Youden index. Additionally, subgroup analysis was conducted based on demographic characteristics such as age, sex, and race, as well as health indicators including overweight status, hypertension, and diabetes. Moreover, given the nonintuitive nature of the significance of AUC increments, the study also employed integrated discrimination improvement (IDI), net weight classification index (NRI) [38, 39], and decision curve analysis (DCA) [40] to further evaluate the findings.

## Results

### Characteristics of the study population

**Fig. 1:**
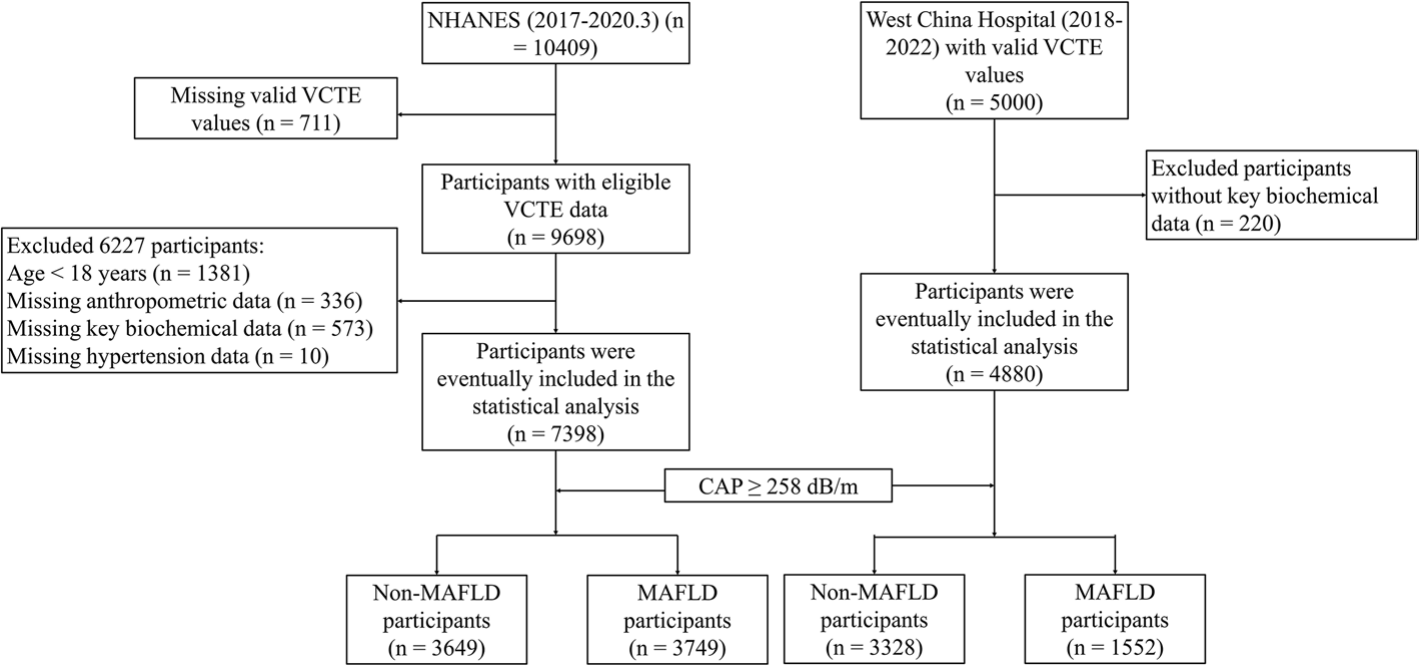
Flow diagram of study design

 Excluding participants without significant variables, a total of 7398 subjects were included in the NHANES cohort from the 10,409 subjects in the 2017-2030.3 NHANES cycle, as depicted in Fig. 1. Likewise, a Western China cohort comprising 4880 patients with valid VCTE and key variables was recruited between 2018 and 2022 at the West China Hospital of Sichuan University. Both cohorts were grouped according to whether they met the diagnostic criteria for MAFLD [5, 6].

Table 1 presents the demographic and clinical features of the two cohorts, namely, the NHANES cohort and the Western China cohort. The NHANES cohort exhibited a MAFLD prevalence of 50.68%, with a mean age of 48.96 ± 18.06 years (45.70 ± 18.99 in the non-MAFLD group and 52.12 ± 16.49 in the MAFLD group). Notably, significant differences in baseline characteristics were observed between participants with and without MAFLD, except for creatinine (CRE). In contrast, the Western China cohort demonstrated a MAFLD prevalence of 31.80%, with a mean age of 44.15 ± 12.14 years (43.64 ± 12.22 in the non-MAFLD group and 45.23 ± 11.89 in the MAFLD group). Similarly, statistically significant differences in all baseline characteristics were observed between participants with and without MAFLD in this cohort.

**Table 1:** Baseline characteristics of participants with or without MAFLD assessed by VCTE in the NHANES cohort and the Western China cohort

Variables	NHANES cohort			Western China cohort		
	Non-MAFLD (*n* = 3649)	MAFLD (*n* = 3749)	*P value*	Non-MAFLD (*n* = 3328)	MAFLD (*n* = 1552)	*P* value
**Demographic parameters**						
Age (years)	45.70 ± 18.99	52.12 ± 16.49	< 0.001	43.64 ± 12.22	45.23 ± 11.89	< 0.001
Sex (%)			< 0.001			< 0.001
Female	1996 (54.70%)	1750 (46.68%)		1639 (49.25%)	303 (19.52%)	
Male	1653 (45.30%)	1999 (53.32%)		1689 (50.75%)	1249 (80.48%)	
Race (%)			< 0.001			-
Non-Hispanic Black	1028 (28.17%)	828 (22.09%)		-	-	
Non-Hispanic White	1235 (33.84%)	1359 (36.25%)		-	-	
Other Hispanic	362 (9.92%)	409 (10.91%)		-	-	
Non-Hispanic Asian	513 (14.06%)	378 (10.08%)		-	-	
Mexican American	331 (9.07%)	596 (15.90%)		-	-	
Other races	180 (4.93%)	179 (4.77%)		-	-	
**Anthropometric parameters**						
WC (cm)	90.51 ± 13.43	109.80 ± 15.01	< 0.001	60.33 ± 9.85	75.30 ± 10.65	< 0.001
WtHR	0.55 ± 0.08	0.66 ± 0.09	< 0.001	0.47 ± 0.05	0.54 ± 0.04	< 0.001
BMI (kg/m^2^)	26.06 ± 5.44	33.35 ± 7.02	< 0.001	22.17 ± 2.55	26.60 ± 2.51	< 0.001
**VCTE parameters**						
CAP (dB/m)	213.26 ± 35.47	314.24 ± 39.31	< 0.001	221.48 ± 25.34	287.23 ± 23.95	< 0.001
LSM (kPa)	5.15 ± 4.10	6.76 ± 5.84	< 0.001	4.93 ± 1.01	5.62 ± 1.11	< 0.001
**Serum test**						
ALT (U/L)	18.83 ± 19.02	25.73 ± 18.38	< 0.001	21.04 ± 15.46	35.72 ± 23.75	< 0.001
AST (U/L)	21.19 ± 15.43	22.56 ± 13.28	< 0.001	21.58 ± 11.37	25.94 ± 12.13	< 0.001
ALP (U/L)	74.46 ± 26.03	81.16 ± 25.37	< 0.001	72.56 ± 21.16	81.03 ± 22.04	< 0.001
GGT (U/L)	26.22 ± 55.77	36.89 ± 46.53	< 0.001	26.56 ± 33.27	48.19 ± 50.57	< 0.001
CRE (umol/L)	78.62 ± 39.12	79.77 ± 41.76	0.223	74.10 ± 15.93	81.50 ± 26.99	< 0.001
eGFR (ml/min/m^2^)	98.05 ± 23.95	93.09 ± 23.18	< 0.001	98.82 ± 14.96	95.56 ± 14.50	< 0.001
UA (umol/L)	298.94 ± 79.40	342.24 ± 87.21	< 0.001	321.70 ± 81.35	387.55 ± 88.18	< 0.001
FPG (umol/L)	5.17 ± 1.27	6.07 ± 2.36	< 0.001	4.93 ± 0.96	5.46 ± 1.52	< 0.001
TG (umol/L)	1.21 ± 0.78	1.86 ± 1.29	< 0.001	1.31 ± 1.25	2.20 ± 1.58	< 0.001
TC (umol/L)	4.70 ± 1.02	4.87 ± 1.06	< 0.001	4.79 ± 0.90	4.96 ± 0.94	< 0.001
HDL (umol/L)	1.50 ± 0.41	1.26 ± 0.36	< 0.001	1.49 ± 0.38	1.18 ± 0.27	< 0.001
**Noninvasive indices and models**						
TyG	8.37 ± 0.54	8.90 ± 0.64	< 0.001	8.36 ± 0.59	8.98 ± 0.62	< 0.001
TyG-BMI	218.59 ± 50.10	296.81 ± 65.24	< 0.001	186.00 ± 29.41	239.05 ± 29.79	< 0.001
TyG-WC	759.51 ± 135.51	977.98 ± 153.37	< 0.001	652.72 ± 102.74	815.28 ± 98.47	< 0.001
TyG-WtHR	4.58 ± 0.84	5.85 ± 0.91	< 0.001	3.96 ± 0.58	4.86 ± 0.56	< 0.001
METS-IR	36.99 ± 9.39	51.46 ± 12.33	< 0.001	31.40 ± 5.79	41.69 ± 6.19	< 0.001
HSI	34.30 ± 6.45	43.78 ± 8.06	< 0.001	30.70 ± 4.13	37.71 ± 4.80	< 0.001
VAI	1.52 ± 1.60	2.81 ± 2.58	< 0.001	1.53 ± 2.79	2.92 ± 2.89	< 0.001
LAP	37.39 ± 33.12	88.53 ± 65.59	< 0.001	23.41 ± 29.28	60.81 ± 50.99	< 0.001
FLI	34.73 ± 28.07	75.70 ± 22.75	< 0.001	18.15 ± 19.20	53.45 ± 24.11	< 0.001
ZJU	36.15 ± 6.24	45.55 ± 7.91	< 0.001	32.21 ± 3.60	38.61 ± 4.03	< 0.001
FSI	-2.00 ± 1.38	0.23 ± 1.67	< 0.001	-2.71 ± 1.24	-0.86 ± 1.42	< 0.001
K-NAFLD	-2.24 ± 2.80	1.14 ± 3.08	< 0.001	-3.31 ± 2.33	0.01 ± 3.24	< 0.001
**Metabolic diseases**						
Hypertension			< 0.001			< 0.001
No	2701 (74.02%)	2015 (53.75%)		3036 (91.23%)	1226 (78.99%)	
Yes	948 (25.98%)	1734 (46.25%)		292 (8.77%)	326 (21.01%)	
Diabetes			< 0.001			< 0.001
No	3324 (91.09%)	2639 (70.39%)		3251 (97.69%)	1429 (92.07%)	
Yes	325 (8.91%)	1110 (29.61%)		77 (2.31%)	123 (7.93%)	

Continuous variables are shown as mean ± SD and compared by Student’s t-test or Mann-Whitney U-test. Categorical values are shown as % and compared using the χ^2^ test

*Abbreviations*: *MAFLD *Metabolic-associated fatty liver disease, *NHANES *National Health and Nutrition Examination Survey, *VCTE *Vibration-controlled transient elastography, *CAP *Controlled attenuation parameter, *LSM *Liver stiffness measurements, *BMI *Body mass index, *WC *Waist circumference, *WtHR *Waist to height ratio, *FPG *Fasting plasma glucose, *ALT *Alanine aminotransferase, *AST *Aspartate aminotransferase, *GGT *γ-glutamyl transpeptidase, *ALP *Alkaline phosphatase, *TC *Total cholesterol, *TG *Triglyceride, *HDL *High-density lipoprotein cholesterol, *UA *Uric acid, *CRE *Creatinine, *eGFR *Estimated glomerular filtration rate

In each of the studied cohorts, it was observed that individuals diagnosed with MAFLD exhibited a greater tendency toward advanced age, male sex, and a higher prevalence of hypertension and diabetes. Additionally, these subjects demonstrated elevated levels of a range of noninvasive indices and models, including METS-IR, TyG, TyG-BMI, TyG-WC, TyG-WtHR, HSI, VAI, FLI, LAP, ZJU, FSI, and K-NAFLD.

### Performances of noninvasive indices and models in predicting MAFLD risk in the NHANES cohort

**Fig. 2:**
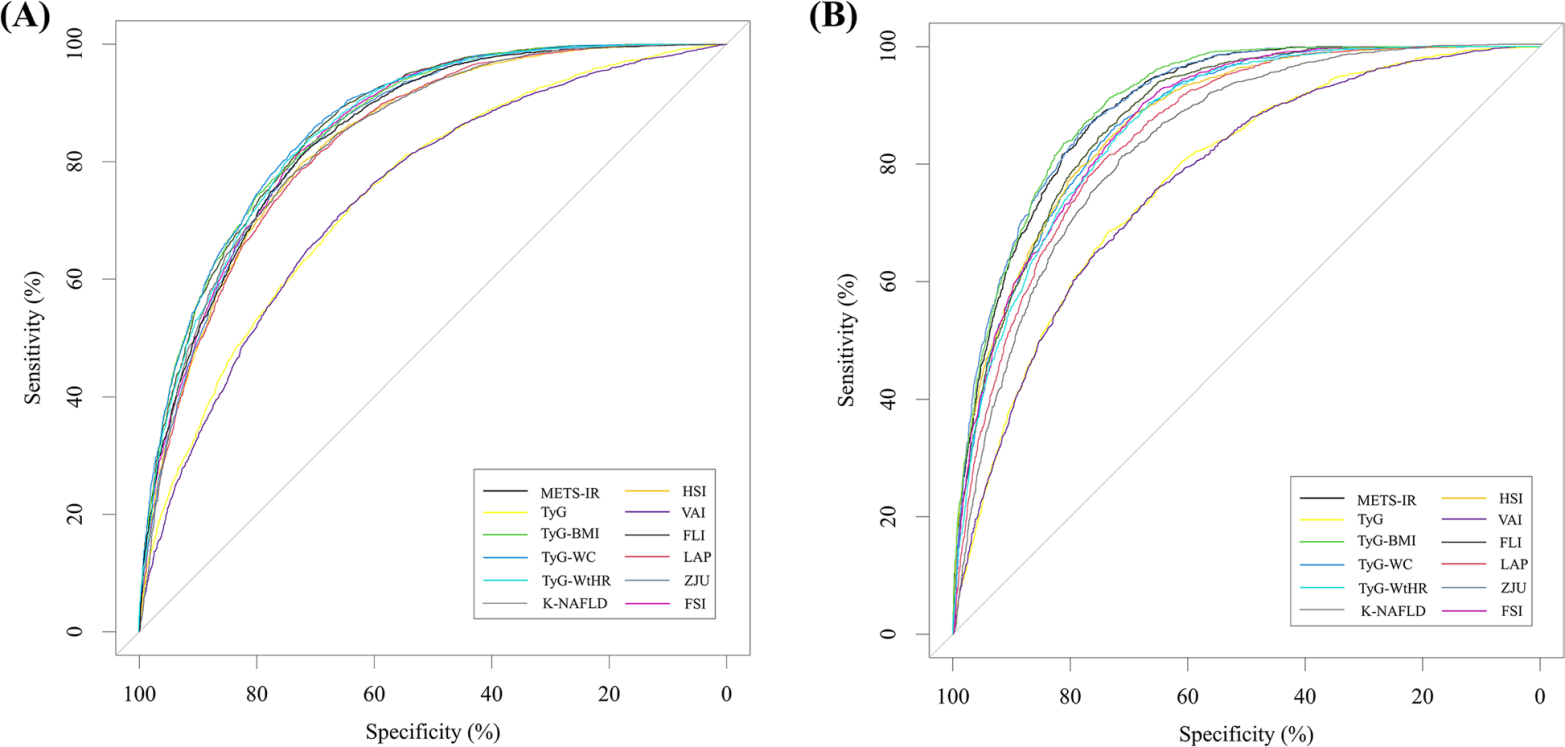
ROC curves for predicting MAFLD in the NHANES cohort (**A**) and Western China cohort (**B**). The x-axis is the specificity; the y-axis is the sensitivity

 The performance of noninvasive indices or models in predicting MAFLD risk in the NHANES cohort was evaluated using ROC curves (Fig. 2A), with AUCs ranging from 0.741 to 0.863. As presented in Table 2, the TyG-WC exhibited the largest AUC (0.863, 95% CI: 0.855–0.871), followed by FLI (0.859 (0.851–0.867)), FSI (0.858 (0.850–0.867)), TyG-WtHR (0.853 (0.845–0.862)), TyG-BMI (0.850 (0.841–0.859)), METS-IR (0.846 (0.837–0.854)), ZJU (0.845 (0.836–0.854)), K-NAFLD (0.838 (0.829–0.847)), HSI (0.835 (0.826–0.844)), LAP (0.834 (0.825–0.843)), TyG (0.746 (0.735–0.757)), and VAI (0.741 (0.730–0.752)). Moreover, an evaluation of 12 noninvasive scores in the NHANES cohort through pairwise comparison demonstrated that TyG-WC exhibited superior predictive performance, as evidenced by a statistically significant difference in AUC compared to the remaining 11 noninvasive scores (all *P* < 0.05) (Supplementary Table 1). There was 70.2% specificity (SPE), 85.9% sensitivity (SEN), 82.9% negative predictive value (NPV), and 74.8% positive predictive value (PPV) for TyG-WC, while the cutoff value was 822.332 Table 3.

**Table 2:** Performance assessment of the noninvasive indices and models for the prediction of MAFLD in the NHANES cohort

Variables	AUC (95% CI)	SEN (95% CI)	SPE (95% CI)	PPV (95% CI)	NPV (95% CI)	Cutoff value
METS-IR	0.846 (0.837–0.854)	0.818 (0.805–0.830)	0.717 (0.703–0.732)	0.748 (0.735–0.761)	0.793 (0.779–0.807)	40.887
TyG	0.746 (0.735–0.757)	0.727 (0.713–0.741)	0.640 (0.625–0.656)	0.675 (0.661–0.689)	0.695 (0.680–0.711)	8.497
TyG-BMI	0.850 (0.841–0.859)	0.819 (0.807–0.831)	0.727 (0.712–0.741)	0.755 (0.741–0.768)	0.796 (0.782–0.810)	240.222
TyG-WC	0.863 (0.855–0.871)	0.859 (0.848–0.870)	0.702 (0.687–0.717)	0.748 (0.735–0.760)	0.829 (0.816–0.842)	822.332
TyG-WtHR	0.853 (0.845–0.862)	0.810 (0.798–0.823)	0.740 (0.726–0.754)	0.762 (0.749–0.775)	0.791 (0.778–0.805)	5.066
HSI	0.835 (0.826–0.844)	0.799 (0.787–0.812)	0.722 (0.708–0.737)	0.747 (0.734–0.761)	0.778 (0.764–0.792)	37.131
VAI	0.741 (0.730–0.752)	0.711 (0.697–0.726)	0.658 (0.643–0.673)	0.681 (0.667–0.696)	0.689 (0.674–0.705)	1.494
FLI	0.859 (0.851–0.867)	0.839 (0.828–0.851)	0.714 (0.700-0.729)	0.751 (0.738–0.764)	0.812 (0.799–0.826)	49.405
LAP	0.834 (0.825–0.843)	0.787 (0.773-0.800)	0.725 (0.711–0.740)	0.746 (0.733–0.760)	0.768 (0.754–0.782)	45.180
ZJU	0.845 (0.836–0.854)	0.826 (0.814–0.838)	0.711 (0.696–0.726)	0.746 (0.733–0.759)	0.799 (0.786–0.813)	38.522
FSI	0.858 (0.850–0.867)	0.768 (0.755–0.782)	0.775 (0.761–0.789)	0.778 (0.765–0.792)	0.765 (0.751–0.779)	-1.068
K-NAFLD	0.838 (0.829–0.847)	0.771 (0.757–0.784)	0.748 (0.734–0.762)	0.759 (0.745–0.772)	0.760 (0.746–0.774)	-1.023

*Abbreviations*: *AUC *Area under the receiver operating characteristic curve, *SPE *Specificity, *SEN *Sensitivity, *NPV *Negative predictive value, *PPV *Positive predictive value

**Table 3:** Performance assessment of the noninvasive indices and models for the prediction of MAFLD in the Western China cohort

Variables	AUC (95% CI)	SEN (95% CI)	SPE (95% CI)	PPV (95% CI)	NPV (95% CI)	Cutoff value
METS-IR	0.896 (0.888–0.905)	0.876 (0.859–0.892)	0.760 (0.745–0.774)	0.630 (0.609–0.650)	0.929 (0.919–0.939)	35.223
TyG	0.776 (0.762–0.789)	0.685 (0.662–0.708)	0.736 (0.721–0.751)	0.547 (0.525–0.570)	0.834 (0.820–0.847)	8.680
TyG-BMI	0.903 (0.895–0.911)	0.836 (0.817–0.854)	0.811 (0.798–0.825)	0.674 (0.653–0.695)	0.914 (0.904–0.924)	211.515
TyG-WC	0.873 (0.864–0.883)	0.865 (0.848–0.882)	0.724 (0.709–0.739)	0.594 (0.574–0.614)	0.920 (0.909–0.930)	714.871
TyG-WtHR	0.866 (0.856–0.876)	0.904 (0.889–0.919)	0.667 (0.651–0.683)	0.559 (0.539–0.578)	0.937 (0.927–0.947)	4.198
HSI	0.873 (0.863–0.883)	0.842 (0.824–0.860)	0.736 (0.721–0.751)	0.598 (0.578–0.619)	0.909 (0.898–0.920)	33.032
VAI	0.773 (0.759–0.786)	0.755 (0.733–0.776)	0.657 (0.641–0.673)	0.506 (0.486–0.527)	0.852 (0.838–0.865)	1.426
FLI	0.879 (0.869–0.888)	0.848 (0.830–0.866)	0.751 (0.736–0.765)	0.613 (0.593–0.634)	0.914 (0.903–0.924)	25.876
LAP	0.854 (0.843–0.864)	0.810 (0.790–0.829)	0.740 (0.725–0.755)	0.592 (0.571–0.613)	0.893 (0.882–0.905)	28.720
ZJU	0.900 (0.891–0.908)	0.865 (0.848–0.882)	0.773 (0.759–0.787)	0.640 (0.620–0.661)	0.925 (0.915–0.935)	34.549
FSI	0.872 (0.863–0.882)	0.904 (0.889–0.919)	0.676 (0.660–0.692)	0.565 (0.546–0.585)	0.938 (0.928–0.948)	-2.408
K-NAFLD	0.836 (0.825–0.847)	0.810 (0.790–0.829)	0.715 (0.700-0.731)	0.570 (0.550–0.591)	0.890 (0.878–0.902)	-2.589

*Abbreviations*: *AUC *Area under the receiver operating characteristic curve, *SPE *specificity, *SEN *Sensitivity, *NPV *Negative predictive value, *PPV *Positive predictive value

To enhance the evaluation of the potential of noninvasive scores in identifying MAFLD risk, NRI and IDI were computed due to the nonintuitive and intricate nature of AUC increments. The outcomes revealed that the NRI and IDI values between TyG-WC and METS-IR/TyG/TyG-BMI/HSI/VAI/LAP/ZJU/FSI/K-NAFLD were greater than 0 and significantly different (all *P* < 0.05). Conversely, the NRI and IDI between TyG-WC and TyG-WtHR/FLI were not statistically significant, as indicated in Supplementary Table 1.

**Fig. 3:**
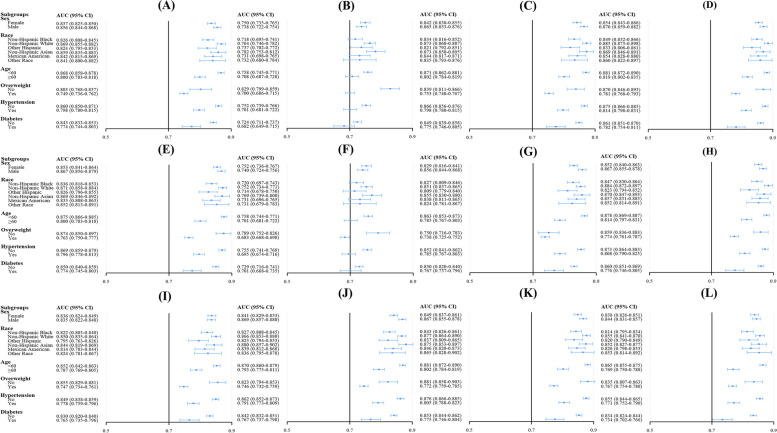
AUC and 95% CI for noninvasive scores to detect MAFLD risk in different subgroups of the NHANES cohort. **A** for METS-IR, (**B**) for TyG, (**C**) for TyG-BMI, (**D**) for TyG-WC, (**E**) for TyG-WtHR, (**F**) for VAI, (**G**) for HSI, (**H**) for FLI, (**I**) for LAP, (**J**) for ZJU , (**K**) for FSI, and (**L**) for K-NAFLD

 The study conducted subgroup analyses by stratifying the participants into distinct subgroups according to variables such as sex, race, age, overweight status, and the presence of diabetes or hypertension. The results, as shown in Fig. 3 and Supplementary Tables 3–14, indicate that the TyG-WC demonstrated the highest AUC among subgroups of male, female, non-Hispanic Black, non-Hispanic White, individuals aged < 60 years, those aged ≥ 60 years, individuals with overweight, individuals with hypertension, individuals without diabetes, and individuals with diabetes. However, in subgroups of Other Hispanic, non-Hispanic Asian, Mexican American, other races, individuals with nonoverweight, and nonhypertensive individuals, the AUC of TyG-WC was not the highest, although the difference was not statistically significant when compared to the noninvasive score with the highest AUC in each subgroup.

**Fig. 4:**
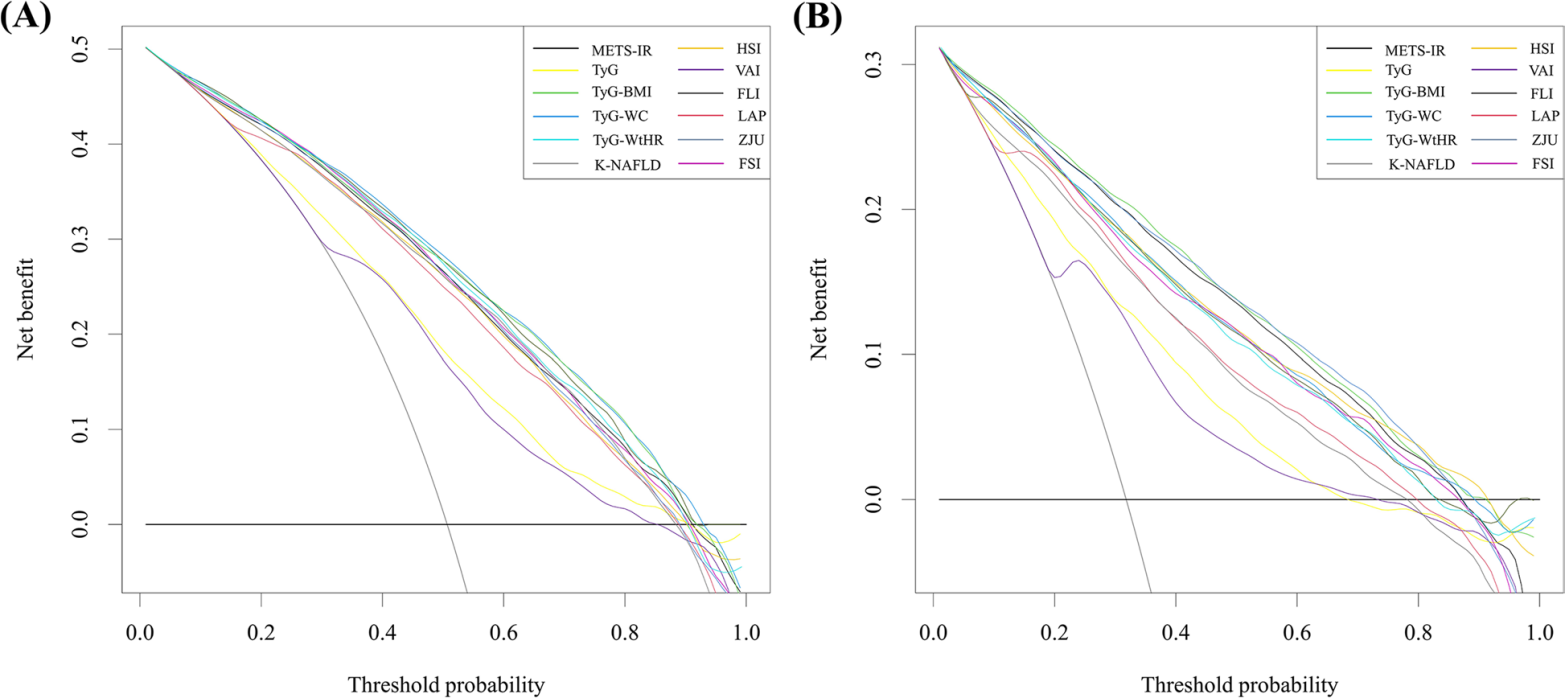
The Clinical utility of the indices were evaluated by decision curves in the NHANES cohort (**A**) and Western China cohort (**B**). The x-axis measures the threshold probability. The y-axis represents net benefits , calculated by subtracting the relative harms (false positive) from the benefits (true positives)

 Furthermore, this study employed DCA to evaluate the clinical usefulness of noninvasive scores by quantifying the probability of net benefit across thresholds ranging from 0.0 to 1.0. The results, as depicted in Fig. 4A, indicate that TyG-WC exhibited a superior net benefit compared to other models within a threshold range of approximately 0.02–0.92, with a maximum net benefit of 0.50.

In summary, the aforementioned findings indicate that the combined predictive value of TyG-WC for MAFLD risk in the NHANES cohort is superior to other indices.

### Performances of noninvasive indices and models in predicting MAFLD risk in the western China cohort

Figure 2B depicts the ROC curves of 12 noninvasive scores for predicting MAFLD risk in the Western China cohort, with AUCs ranging from 0.773 to 0.903. Notably, unlike the NHANES cohort, the TyG-BMI score exhibited the largest AUC (0.903; 95% CI: 0.895–0.911), followed by ZJU (0.900 (0.891–0.908)), METS-IR (0.896 (0.888–0.905)), FLI (0.879 (0.869–0.888)), TyG-WC (0. 873 (0.864–0.883)), HSI (0.873 (0.863–0.883)), FSI (0.872 (0.863–0.882)), TyG-WtHR (0.866 (0.856–0.876)), LAP (0.854 (0.843–0.864)), K-NAFLD (0.836 (0.825–0.847)), TyG (0.776 (0.762–0.789)), and VAI (0.773 (0.759–0.786)). Upon conducting additional pairwise comparisons of AUC differences, it was determined that TyG-BMI exhibited statistically significant differences from the remaining 10 noninvasive scores (all *P* < 0.05), with the exception of ZJU (*P* = 0.145) (Supplementary Table 2). The SPE for TyG-BMI was 81.1%, SEN was 83.6%, NPV was 91.4%, PPV was 67.4%, and the critical value was 211.515. Furthermore, the NRI and IDI values of TyG-BMI in comparison to the remaining nine noninvasive scores were observed to be greater than 0 and exhibited a significant difference (*P* < 0.05), with the exception of METS-IR and ZJU, as evidenced in Supplementary Table 2. Consistently, the results of DCA curves, as shown in Fig. 4B, indicated that TyG-BMI possessed greater net benefits compared to the other models within the threshold range of approximately 0.01 to 0.91, with a maximum net benefit of 0.31.

**Fig. 5:**
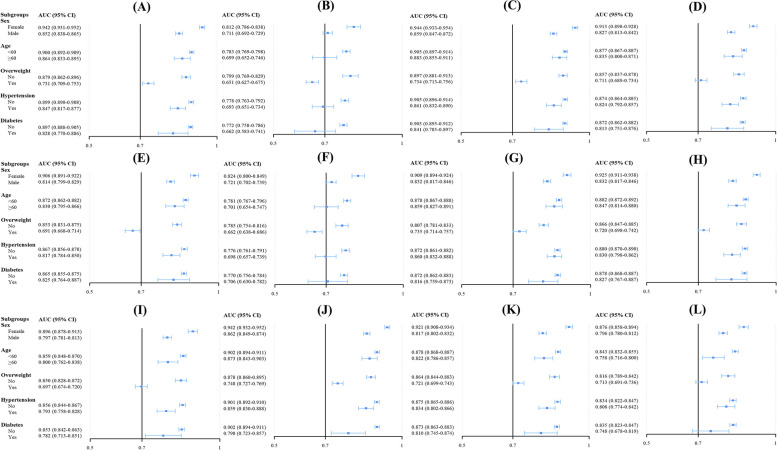
AUC and 95% CI for noninvasive scores to detect MAFLD risk in different subgroups of the Western China cohort. **A** for METS-IR, (**B**) for TyG, (**C**) for TyG-BMI, (**D**) for TyG-WC, (**E**) for TyG-WtHR, (**F**) for VAI, (**G**) for HSI, (**H**) for FLI, (**I**) for LAP, (**J**) for ZJU, (**K**) for FSI, and (**L**) for K-NAFLD

 In the subgroup analysis, as shown in Fig. 5 and Supplementary Tables 15–26, TyG-BMI exhibited the highest AUC in the Western China cohort across various subgroups, including women, individuals under the age of 60, those aged 60 years or older, individuals with nonoverweight, and those who were nonhypertensive and hypertensive, nondiabetic and diabetic. In the male subgroup, ZJU demonstrated the highest AUC, although the difference in AUC between ZJU and TyG-BMI was not statistically significant (*P* = 0.504). Additionally, ZJU exhibited the highest AUC in the overweight subgroup, without a statistically significant difference in AUC compared to TyG-BMI (*P* = 0.308).

Overall, the above results suggest that TyG-BMI was superior to other indices in terms of its combined ability to predict MAFLD risk in the Western China cohort.

## Discussion

MAFLD is the most prevalent chronic liver disease globally, and its disease burden is on the rise. Consequently, numerous studies are investigating noninvasive, pragmatic, and dependable disease predictive models to identify and manage individuals at high risk of MAFLD, ultimately mitigating the disease burden. The present study evaluated the predictive capacity of 12 widely employed, noninvasive indices or models for estimating the risk of MAFLD in both U.S. and Chinese cohorts. The predictive capacity of TyG and VAI in determining individual risk for MAFLD was found to be limited in both the NHANES and Western China cohorts, whereas the remaining 10 noninvasive scores demonstrated superior predictive performance. It is noteworthy that although variations in predictive performance were observed across different populations and subgroups, the TyG-related indices exhibited superior performance in predicting MAFLD overall. Specifically, the TyG-WC demonstrated the best performance in the NHANES cohort, while the TyG-BMI exhibited the best performance in the Western China cohort. Despite not achieving the highest AUC in certain subgroups, the lack of statistical significance in the difference between the AUC of TyG-WC/TyG-BMI and the highest AUC within the corresponding subgroups, combined with the straightforward calculation formula, minimal variables needed, and cost-effectiveness, suggests that TyG-WC/TyG-BMI outperformed other methods overall.

MAFLD is a multifactorial disease of a complex nature that encompasses genetics, environment, and metabolism [41]. Among them, genetic susceptibility genes associated with MAFLD have received attention from researchers in recent years, especially transmembrane 6 superfamily member 2 (TM6SF2) [42]. TM6SF2 plays a crucial role in hepatocellular lipid metabolism, regulates very low-density lipoprotein (VLDL) secretion and is implicated in hepatocyte inflammation [43]. The nonsynonymous variant in TM6SF2 (E167K, rs58542926) results in protein dysfunction, leading to an excessive buildup of TG in the liver, thereby contributing to the onset of fatty liver disease [44]. However, the pathogenesis of MAFLD remains incompletely understood, with the “double-hit” and “multiple-hit” theories being the most widely accepted [45, 46]. Regardless of the “double-hit” theory or “multiple-hit” theory, IR is a crucial component in the development of MAFLD [8, 47–51]. At present, the hyperinsulinemic-euglycemic clamp (HEC) [52, 53] technique stands as the foremost method for directly assessing IR. However, owing to its operational intricacy, noninvasive fasting insulin (FINS)-based indices are commonly employed in clinical settings to evaluate IR. Nonetheless, the clinical utility of FINS is constrained due to its nonroutine nature. Consequently, a plethora of fasting insulin-independent indices, including TyG-related indices and METS-IR, have been devised to accurately reflect IR and serve as indirect substitutes [10–13, 20]. The mechanism may be attributed to the two primary constituents of TyG-WC/TyG-BMI (TG and FPG), which are associated with “glucotoxicity” and “lipotoxicity” that play a key role in the development of IR [54, 55]. TGs are predominantly derived from the uptake of free fatty acids (FFAs) by the liver. In the presence of IR, the breakdown of adipose tissue in the periphery is augmented, resulting in an excessive production of FFA that enters the liver via the portal vein system and accumulates aberrantly within the hepatic tissue. Consequently, this abnormal accumulation leads to an upregulation of intrahepatic TG synthesis. Moreover, elevated concentrations of FFAs exhibit lipotoxic properties, impeding insulin signaling and impairing insulin utilization in various target organs across the body, thereby exacerbating IR. It is noteworthy that the excessive influx of FFAs into skeletal muscle and hepatocytes hampers glucose uptake in these tissues by inhibiting insulin, thereby disrupting glucose metabolism [56, 57]. It is evident that IR significantly influences the onset and progression of NAFLD/MAFLD by disrupting glucose and lipid metabolism and promoting excessive fat accumulation in hepatocytes. This reciprocal relationship between IR and hepatocellular fat storage establishes a detrimental cycle that constantly promotes the development and advancement of NAFLD/MAFLD. Since the two components of TyG-BMI/TyG-WC are important components of glucose metabolism and lipid metabolism, respectively, TyG-WC/TyG-BMI is closely related to the occurrence of MAFLD, which is not only a risk factor for MAFLD but also a reliable indicator for the prediction of MAFLD. Considering the favorable predictive capabilities of TyG-BMI/TyG-WC in assessing the risk of MAFLD, it would be advantageous to prioritize the inclusion of metrics pertaining to IR, lipid metabolism, and glucose metabolism, such as FPG, TG, and the emerging focus on remnant cholesterol (RC), in the development of future MAFLD prediction models. RC predominantly signifies cholesterol content within VLDL remnants and exhibits a significant association with the risk of MAFLD/NAFLD [58, 59].

Prior research has demonstrated the favorable predictive capacity of TyG-related indices in relation to NAFLD/MAFLD [14–19, 60–62]. Khamseh et al. [60] found that TyG-WC and TyG-BMI were significantly associated with NAFLD in an overweight/obese cohort and could reliably predict the risk of NAFLD in this population. In addition, Sheng et al. [61] conducted a comparative analysis of 15 indices related to obesity and lipid levels, revealing that TyG-related parameters exhibited the most robust association with NAFLD. Specifically, in the female subgroup, TyG-WC demonstrated a predictive capacity for NAFLD with an AUC of 0.905, while TyG-BMI exhibited an AUC of 0.908. In the male subgroup, TyG-WC displayed an AUC of 0.836, and TyG-BMI displayed an AUC of 0.843. Furthermore, Chang and colleagues [62] investigated the prognostic efficacy of TyG-associated indices for MAFLD in 20,922 Chinese participants. The results indicated that TyG-BMI exhibited the highest predictive capacity, with an AUC of 0.933 (0.927–0.938) in the female subgroup and 0.870 (0.864–0.876) in the male subgroup. TyG-WC followed with AUCs of 0.922 (0.915–0.928) and 0.847 (0.841–0.854), respectively. A paucity of research has conducted comparisons between predictive models developed for fatty liver and indirect indices that reflect IR. A study utilizing the NHANES database conducted a comparison between the TyG-related indices and VAI and LAP, revealing that the former exhibited superior predictive capabilities for both MAFLD and NAFLD risk [19]. The studies mentioned earlier were assessed using a singular approach, relying solely on the AUC to determine predictive value. In contrast, the current study stands out as the most extensive investigation of predictive models for fatty liver and noninvasive indices that indirectly reflect IR in a cross-sectional analysis. Furthermore, this study systematically and comprehensively evaluates the predictive capacity of these models.

In the NHANES cohort, the AUC of FLI was found to be greater than that of other noninvasive scores, with the exception of TyG-WC. Similarly, in the Chinese cohort, the AUC of ZJU was observed to be higher than that of other noninvasive scores, except for TyG-BMI. The findings were consistent with prior external validation studies of fatty liver prediction models. Li et al. conducted an external validation of ZJU, FLI, HSI, LAP, and VAI for NAFLD risk in a cohort of 19,804 individuals in western China and reported that ZJU exhibited an AUC of 0.925 (95% CI: 0.919–0.931) with a cutoff value of 35.29, surpassing the performance of the other four noninvasive models. Furthermore, ZJU demonstrated superior sensitivity, specificity, positive predictive value, and negative predictive value compared to the other four models [63]. Fu et al. conducted a study on 107 severely obese Western women with NAFLD and found that ZJU outperformed HSI, LAP, and VAI, with an AUC of 0.742 (95% 0.647–0.837) [64]. A Japanese study revealed that ZJU and FLI had similar AUCs of 0.886 and 0.884, respectively. Further analysis by sex indicated that ZJU had a higher AUC than FLI in both the male and female groups, while FLI performed better in the diabetes subgroup [65]. However, in a study from eastern China, FLI demonstrated a superior AUC of 0.852 (95% 0.839–0.864) for NAFLD risk compared to ZJU, LAP, and VAI, and DCA showed a higher net benefit [66].

After the renaming of NAFLD to MAFLD, a limited number of studies have investigated the efficacy of predictive models in diagnosing MAFLD. Notably, a recent study utilizing the NHANES III database found that FLI exhibited the highest diagnostic value for MAFLD diagnosed by ultrasonography, with an AUC of 0.793 (0.786-0.800) [67]. Additionally, an external validation article assessing MAFLD diagnosed by VCTE, also based on the NHANES database, reported that FLI had a superior AUC of 0.840 (95% 0.822–0.858) compared to ZJU (0.826 (0.808–0.845)), FSI (0.833 (0.815–0.852)), HSI (0.814 (0.795–0.834)), LAP (0.826 (0.807–0.844)), and VAI (0.747 (0.723–0.770)) [68]. Furthermore, Han and colleagues conducted an analysis of noninvasive prediction models for the diagnosis of MAFLD using CT and determined that FLI exhibited the most effective diagnostic ability, with the highest AUC of 0.791 (95% 0.766–0.816) and an optimal cutoff value of 29.9, which was better than HSI, VAI, ZJU, and LAP [69]. The ZJU algorithm, developed in China, comprises BMI, FPG, TG, and ALT/AST ratio, while the FLI algorithm, first developed in Italy in 2006, is the first predictive model applied to the diagnosis of NAFLD, consisting of BMI, TG, GGT, and WC. Both algorithms incorporate variables that reflect metabolic conditions, which are a crucial aspect emphasized in the diagnosis of MAFLD. This may explain the superior performance of FLI and ZJU in identifying MAFLD risk. In the Western China population, ZJU exhibited superior performance compared to FLI. This trend was further substantiated in the subgroup analysis of the study, wherein the NHANSE cohort indicated that although FLI outperformed ZJU overall, ZJU exhibited the highest AUC of 0.880 (95% 0.857–0.902) among the non-Hispanic Asian group (Supplement Table 12).

Recently, several hepatology societies have gone through several rounds of investigation and discussion to form a consensus [70]. Specifically, they have proposed the adoption of a novel terminology for the precise categorization and nomenclature of fatty liver disease. Under this proposed framework, steatotic liver disease (SLD) would serve as a comprehensive term encompassing the diverse etiologies of steatosis, which include metabolic dysfunction-associated steatotic liver disease (MASLD), metabolic and alcohol-related steatotic liver disease (MetALD), alcohol-associated liver disease (ALD), etiology-specific SLD, and cryptogenic SLD. The diagnosis of MASLD bears striking resemblance to MAFLD but requires only the presence of one cardiovascular-related metabolic disorder and hepatic steatosis to be diagnosed. Overall, the release of the consensus is only a first step, and the impact of the new disease name and diagnostic criteria on other populations and organizations needs to be further evaluated. Irrespective of the alteration in nomenclature, it is critical that noninvasive methods identify people at high risk for fatty liver disease early and determine the point at which specialized treatment is needed.

### Study strengths and limitations

Several advantages of this study are worth mentioning. First, in this research, liver steatosis was measured by VCTE, which is more accurate than ultrasonography [71]. Second, this study comprised two validation cohorts consisting of 12,278 participants from the United States and China. The inclusion of such a substantial sample size and data from two different centers enhances the reliability of the study’s findings. Third, this study aimed to assess and compare noninvasive indices and models in terms of their AUC, subgroup analysis, NRI, IDI, and DCA, thus providing a more comprehensive analysis compared to previous literature.

On the other hand, there are some limitations. First, due to its impracticality and invasiveness in a sample of thousands, liver biopsy, the diagnostic gold standard, was not performed in this study. Second, this study found that the optimal noninvasive model for MAFLD risk identification was different across regions and races; therefore, external validation is needed in regions other than China and the United States.

## Conclusion

In summary, TyG-BMI demonstrated satisfactory diagnostic efficacy in identifying individuals at high risk of MAFLD in the western Chinese population, surpassing other noninvasive scores or models. Conversely, TyG-WC exhibited optimal diagnostic value and satisfactory diagnostic performance for high-risk MAFLD in the US population. These indices necessitate fewer variables, possess straightforward calculation formulas, are cost-effective, and can be applied across various medical institutions to facilitate early identification, treatment, and mitigation of the disease burden. In light of the findings obtained from the present study, it may be advisable to discontinue the pursuit of a “perfect” noninvasive model and instead employ the most appropriate model tailored to different regions and ethnicities.

### Supplementary Information

**Additional file 1.**

## Data Availability

Data pertaining to NHANES can be accessed on its official website (https://www.cdc.gov/nchs/nhanes/).

## Abbreviations

MAFLD: Metabolic-associated fatty liver disease
NAFLD: Nonalcoholic fatty liver disease
SLD: Steatotic liver disease
MASLD: Metabolic dysfunction associated steatotic liver disease
MetALD: Metabolic and alcohol-related steatosis liver disease
ALD: Alcohol-associated liver disease
APASL: Asia-Pacific Association for the Study of the Liver
KNHANES: Korean National Health and Nutrition Examination Survey
NHANES: National Health and Nutrition Examination Survey
NCHS: National Center for Health Statistics
TRIPOD: Transparent Reporting of a Multivariable Predictive Model for Individual Prognosis or Diagnosis
CT: Computed tomography
VCTE: Vibration-controlled transient elastography
CAP: Controlled attenuation parameter
LSM: Liver stiffness measurements
IR: Insulin resistance
HEC: Hyperinsulinemic-euglycemic clamp
FINS: Fasting insulin
BMI: Body mass index
WC: Waist circumference
WtHR: Waist to height ratio
ALT: Alanine aminotransferase
AST: Aspartate aminotransferase
GGT: γ-glutamyl transpeptidase
ALP: Alkaline phosphatase
TC: Total cholesterol
TG: Triglyceride
TM6SF2: Transmembrane 6 superfamily member 2
FFA: Free fatty acids
VLDL: Very low-density lipoprotein
RC: Remnant cholesterol
HDL: High-density lipoprotein cholesterol
UA: Uric acid
CRE: Creatinine
eGFR: Estimated glomerular filtration rate
ROC: Receiver operating characteristic curve
SEN: Sensitivity
SPE: Specificity
PPV: Positive predictive value
NPV: Negative predictive value
AUC: Area under receiver operating characteristic curve
NRI: Net reclassification index
IDI: Integrated discrimination improvement
DCA: Decision curve analysis
OR: Odds ratio
CI: Confidence interval
FLI: Fatty liver index
FSI: Framingham steatosis index
HSI: Hepatic steatosis index
VAI: Visceral adiposity index
ZJU: Zhejiang University index
LAP: Lipid accumulation product
TyG: Triglyceride glucose index
TyG-BMI: Triglyceride glucose-body mass index
TyG-WC: Triglyceride glucose-waist circumference index
TyG-WtHR: Triglyceride glucose-waist to height ratio index
METS-IR: Metabolic score for insulin resistance
