# Supplementary material for: Comparison of the diagnostic performance of twelve noninvasive scores of metabolic dysfunction-associated fatty liver disease
Source: Lipids Health Dis. 2023 Sep 6;22:145. doi: 10.1186/s12944-023-01902-3 (PMC10481547; doi:10.1186/s12944-023-01902-3)
Supplement: Supplementary file 1 — Additional file 1. [file 12944_2023_1902_MOESM1_ESM.docx]

**Supplementary Material**

**1.Laboratory measurement and clinical data**

The following variables were obtained from the original NHANES and the Western China dataset: demographic parameters (age, sex, and race (only for NHANES dataset)), anthropometric parameters (waist circumference (WC), height, waist to height ratio (WtHR), body mass index (BMI)), vibration-controlled transient elastography (VCTE) parameters (liver stiffness measurements (LSM) and controlled attenuation parameter (CAP)), comorbidities (hypertension and diabetes), and biomarkers such as fasting plasma glucose (FPG), triglyceride (TG), alanine aminotransferase (ALT), aspartate aminotransferase (AST), alkaline phosphatase (ALP), γ-glutamyl transpeptidase (GGT), high-density lipoprotein cholesterol (HDL), total lipoprotein cholesterol (TC), uric acid (UA), creatinine (CRE), estimated glomerular filtration rate (eGFR).

We categorized race into six groups (other Hispanic, non-Hispanic Asian, non-Hispanic Black, non-Hispanic White, Mexican American, and other races) (only for NHANES dataset).

The diagnostic criteria for overweight/obesity were BMI ≥ 25/30 kg/m^2^ in NHANES cohort and BMI ≥ 23/27.5 kg/m^2^ in Western China cohort [1, 2]. The diagnostic criteria for diabetes were glycohemoglobin (HbA1c) > 6.5% or random plasma glucose ≥ 11.1 mmol/L or FPG ≥ 7.0 mmol/L or two-hour oral glucose tolerance test (OGTT) plasma glucose ≥ 11.1 mmol/L or under anti-diabetes therapy, or self-reported diabetes [3]. The definition of hypertension was based on systolic blood pressure (SBP) greater than 140 mmHg or diastolic blood pressure (DBP) greater than 90 mmHg, self-reported hypertension, or under antihypertension treatment [4].

Formulas for calculating METS-IR [5], TyG [6], TyG-BMI [7], TyG-WC [8], and TyG-WtHR [9] were as follows: METS-IR = Ln [2 × FPG (mg/dL) + TG (mg/dL)] × BMI (kg/m^2^) / Ln [HDL (mg/dL)]; TyG = Ln [TG (mg/dL) × FPG (mg/dL) / 2]; TyG-BMI = Ln [TG (mg/dL) × FPG (mg/dL) / 2] × BMI (kg/m^2^); TyG-WC = Ln [TG (mg/dL) × FPG (mg/dL) / 2] × WC (cm); and TyG-WtHR = Ln [TG (mg/dL) × FPG (mg/dL) / 2] × [WC (cm) / Height (cm)].

The equations for HSI [10], VAI [11], FLI [12], LAP [13], ZJU [14], FSI [15]were as follows: HSI = 8 × [ALT (U/L) / AST (U/L)] + BMI (kg/m^2^) (+ 2, if diabetes; + 2, if female); VAI = [WC (cm) / (39.68 + 1.88 × BMI (kg/m^2^))] × [TG (mmol/L) / 1.03] × [1.31 / HDL (mmol/L)], for male; [WC (cm) / (36.58 + 1.89 × BMI (kg/m^2^))] × (TG (mmol/L) / 0.81) × (1.52 / HDL (mmol/L)), for female; FLI = (e ^0.953 × ln TG (mg/dL) + 0.139 × BMI (kg/m2) + 0.718 × ln GGT (U/L) + 0.053 × WC - 15.745)^/(1 +e ^0.953 × ln TG (mg/dL) + 0.139 × BMI (kg/m2) + 0.718 × ln GGT (U/L) + 0.053 × WC - 15.745)^) × 100; LAP = [WC (cm) - 65] × TG (mmol/L) for males, LAP = [WC (cm) - 58] × TG (mmol/L) for females; ZJU = BMI (kg/m^2^) + FPG (mmol/L) + TG (mmol/L) + 3 × [ALT (U/L) / AST (U/L)] (+ 2, if female); FSI = -7.981 + 0.011 × Age (years) - 0.146 × Sex (female = 1, male = 0) + 0.173 × BMI (kg/m^2^) + 0.007 × TG (mg/dL) + 0.593 × Hypertension (yes = 1, no = 0) + 0.789 × Diabetes (yes = 1, no = 0) + 11 × [ALT (U/L) / AST (U/L) > 1.33 (yes = 1, no = 0)]; and K-NAFLD = 0.913× Sex (2, if female; 1, if male) + 0.089 × WC (cm) + 0.032 × [SBP (mmHg) + FPG (mg/dL)] + TG (mg/dL) × 0.007 + ALT (U/L) × 0.105 - 20.929 [16].

**2.The definition of MAFLD**

MAFLD was defined as the presence of hepatic steatosis with one or more of the following [1, 17, 18]:

1). Overweight or obesity;

2). Diabetes;

3). All of the following items at least meet two metabolic abnormalities:

a. WC ≥102 cm in men and ≥88 cm in women;

b. Blood pressure ≥ 130/85 mmHg;

c. TG ≥ 1.70 mmol/L;

d. HDL < 1.0 mmol/L for men and <1.3 mmol/L for women;

e. Prediabetes (i.e., FPG 5.6 to 6.9 mmol/L, or HbA1c 5.7% to 6.4%);

f. HOMA-IR score ≥2.5;

g. hsCRP ≥2 mg/L.

**Referrences**

1. Eslam M, Sarin SK, Wong VW, Fan JG, Kawaguchi T, Ahn SH, Zheng MH, Shiha G, Yilmaz Y, Gani R, et al: **The Asian Pacific Association for the Study of the Liver clinical practice guidelines for the diagnosis and management of metabolic associated fatty liver disease.** *Hepatol Int* 2020, **14:**889-919.

2. **Appropriate body-mass index for Asian populations and its implications for policy and intervention strategies.** *Lancet* 2004, **363:**157-163.

3. American Diabetes A: **Classification and Diagnosis of Diabetes: Standards of Medical Care in Diabetes—2020.** *Diabetes Care* 2019, **43:**S14-S31.

4. Williams B, Mancia G, Spiering W, Agabiti Rosei E, Azizi M, Burnier M, Clement DL, Coca A, de Simone G, Dominiczak A, et al: **2018 ESC/ESH Guidelines for the management of arterial hypertension.** *Eur Heart J* 2018, **39:**3021-3104.

5. Bello-Chavolla OY, Almeda-Valdes P, Gomez-Velasco D, Viveros-Ruiz T, Cruz-Bautista I, Romo-Romo A, Sánchez-Lázaro D, Meza-Oviedo D, Vargas-Vázquez A, Campos OA, et al: **METS-IR, a novel score to evaluate insulin sensitivity, is predictive of visceral adiposity and incident type 2 diabetes.** *Eur J Endocrinol* 2018, **178:**533-544.

6. Simental-Mendía LE, Rodríguez-Morán M, Guerrero-Romero F: **The Product of Fasting Glucose and Triglycerides As Surrogate for Identifying Insulin Resistance in Apparently Healthy Subjects.** *Metabolic Syndrome and Related Disorders* 2008, **6:**299-304.

7. Er LK, Wu S, Chou HH, Hsu LA, Teng MS, Sun YC, Ko YL: **Triglyceride Glucose-Body Mass Index Is a Simple and Clinically Useful Surrogate Marker for Insulin Resistance in Nondiabetic Individuals.** *PLoS One* 2016, **11:**e0149731.

8. Zheng S, Shi S, Ren X, Han T, Li Y, Chen Y, Liu W, Hou PC, Hu Y: **Triglyceride glucose-waist circumference, a novel and effective predictor of diabetes in first-degree relatives of type 2 diabetes patients: cross-sectional and prospective cohort study.** *J Transl Med* 2016, **14:**260.

9. Lim J, Kim J, Koo SH, Kwon GC: **Comparison of triglyceride glucose index, and related parameters to predict insulin resistance in Korean adults: An analysis of the 2007-2010 Korean National Health and Nutrition Examination Survey.** *PLoS One* 2019, **14:**e0212963.

10. Lee J, Kim D, Kim H, Lee C, Yang J, Kim W, Kim Y, Yoon J, Cho S, Sung M, Lee HJ: **Hepatic steatosis index: a simple screening tool reflecting nonalcoholic fatty liver disease.** *Digestive and Liver Disease* 2010, **42:**503-508.

11. Amato MC, Giordano C, Galia M, Criscimanna A, Vitabile S, Midiri M, Galluzzo A, Group ftAS: **Visceral Adiposity Index: A reliable indicator of visceral fat function associated with cardiometabolic risk.** *Diabetes Care* 2010, **33:**920-922.

12. Bedogni G, Bellentani S, Miglioli L, Masutti F, Passalacqua M, Castiglione A, Tiribelli C: **The Fatty Liver Index: a simple and accurate predictor of hepatic steatosis in the general population.** *BMC Gastroenterology* 2006, **6:**33.

13. Kahn HS: **The lipid accumulation product is better than BMI for identifying diabetes: a population-based comparison.** *Diabetes Care* 2006, **29:**151-153.

14. Wang J, Xu C, Xun Y, Lu Z, Shi J, Yu C, Li YJ: **ZJU index: a novel model for predicting nonalcoholic fatty liver disease in a Chinese population.** *Scientific reports* 2015, **5:**16494.

15. Long MT, Pedley A, Colantonio LD, Massaro JM, Hoffmann U, Muntner P, Fox CS: **Development and Validation of the Framingham Steatosis Index to Identify Persons With Hepatic Steatosis.** *Clinical Gastroenterology and Hepatology* 2016, **14:**1172-1180.e1172.

16. Jeong S, Kim K, Chang J, Choi S, Kim SM, Son JS, Lee G, Kim W, Park SM: **Development of a simple nonalcoholic fatty liver disease scoring system indicative of metabolic risks and insulin resistance.** *Ann Transl Med* 2020, **8:**1414.

17. Eslam M, Newsome PN, Sarin SK, Anstee QM, Targher G, Romero-Gomez M, Zelber-Sagi S, Wai-Sun Wong V, Dufour J-F, Schattenberg JM, et al: **A new definition for metabolic dysfunction-associated fatty liver disease: An international expert consensus statement.** *Journal of Hepatology* 2020, **73:**202-209.

18. Eslam M, Sanyal AJ, George J: **MAFLD: A Consensus-Driven Proposed Nomenclature for Metabolic Associated Fatty Liver Disease.** *Gastroenterology* 2020, **158:**1999-2014.e1991.

**3.Supplementary Tables**

**Supplement Table 1** Performance assessment of the noninvasive indices and models for the prediction of MAFLD in the NHANES cohort

| Pairwise comparison | Difference AUC (95% CI) | *P* value | NRI (95% CI) | *P* value | IDI (95% CI) | *P* value |
| --- | --- | --- | --- | --- | --- | --- |
| METS-IR vs. TyG | 0.099 (0.088-0.111) | <0.001 | 0.168 (0.143-0.193) | <0.001 | 0.154 (0.131-0.177) | <0.001 |
| TyG-BMI vs. METS-IR | 0.004 (0.002-0.007) | 0.001 | 0.011 (-0.001-0.023) | 0.065 | 0.011 (-0.006-0.028) | 0.206 |
| TyG-WC vs. METS-IR | 0.018 (0.013-0.022) | <0.001 | 0.026 (0.010-0.042) | 0.002 | 0.034 (0.014-0.054) | 0.001 |
| TyG-WtHR vs. METS-IR | 0.008 (0.003-0.013) | 0.004 | 0.015 (-0.002-0.032) | 0.087 | 0.015 (-0.006-0.036) | 0.155 |
| METS-IR vs. HSI | 0.010 (0.005-0.015) | <0.001 | 0.013 (-0.003-0.029) | 0.114 | 0.015 (-0.005-0.035) | 0.137 |
| METS-IR vs. VAI | 0.104 (0.094-0.115) | <0.001 | 0.166 (0.142-0.190) | <0.001 | 0.153 (0.131-0.175) | <0.001 |
| FLI vs. METS-IR | 0.014 (0.009-0.018) | <0.001 | 0.019 (0.003-0.035) | 0.017 | 0.023 (0.003-0.043) | 0.023 |
| METS-IR vs. LAP | 0.012 (0.004-0.019) | 0.002 | 0.023 (0.004-0.042) | 0.019 | 0.026 (0.005-0.047) | 0.018 |
| METS-IR vs. ZJU | 0.001 (-0.003-0.004) | 0.770 | -0.003 (-0.017-0.011) | 0.678 | -0.004 (-0.023-0.015) | 0.675 |
| FSI vs. METS-IR | 0.013 (0.008-0.018) | <0.001 | 0.008 (-0.010-0.026) | 0.373 | 0.005 (-0.016-0.026) | 0.637 |
| METS-IR vs. K-NAFLD | 0.007 (-0.001-0.016) | 0.070 | 0.016 (-0.005-0.037) | 0.131 | 0.020 (-0.002-0.042) | 0.080 |
| TyG-BMI vs. TyG | 0.104 (0.092-0.115) | <0.001 | 0.179 (0.154-0.204) | <0.001 | 0.165 (0.142-0.188) | <0.001 |
| TyG-WC vs. TyG | 0.117 (0.107-0.127) | <0.001 | 0.194 (0.171-0.216) | <0.001 | 0.188 (0.166-0.210) | <0.001 |
| TyG-WtHR vs. TyG | 0.107 (0.097-0.117) | <0.001 | 0.183 (0.160-0.203) | <0.001 | 0.169 (0.147-0.191) | <0.001 |
| HSI vs. TyG | 0.089 (0.076-0.102) | <0.001 | 0.155 (0.128-0.182) | <0.001 | 0.138 (0.115-0.161) | <0.001 |
| TyG vs. VAI | 0.005 (-0.001-0.011) | 0.083 | -0.003 (-0.020-0.014) | 0.723 | -0.001 (-0.018-0.016) | 0.907 |
| FLI vs. TyG | 0.113 (0.102-0.124) | <0.001 | 0.187 (0.163-0.211) | <0.001 | 0.177 (0.154-0.200) | <0.001 |
| LAP vs. TyG | 0.088 (0.081-0.095) | <0.001 | 0.145 (0.126-0.164) | <0.001 | 0.128 (0.108-0.148) | <0.001 |
| ZJU vs. TyG | 0.099 (0.087-0.111) | <0.001 | 0.171 (0.145-0.197) | <0.001 | 0.158 (0.135-0.181) | <0.001 |
| FSI vs. TyG | 0.112 (0.102-0.122) | <0.001 | 0.176 (0.142-0.190) | <0.001 | 0.159 (0.137-0.181) | <0.001 |
| K-NAFLD vs. TyG | 0.092 (0.081-0.102) | <0.001 | 0.152 (0.128-0.176) | <0.001 | 0.134 (0.112-0.156) | <0.001 |
| TyG-WC vs. TyG-BMI | 0.013 (0.009-0.018) | <0.001 | 0.016 (0.001-0.031) | 0.042 | 0.023 (0.003-0.043) | 0.023 |
| TyG-WtHR vs.TyG-BMI | 0.003 (-0.001-0.008) | 0.125 | 0.004 (-0.011-0.019) | 0.608 | 0.004 (-0.016-0.024) | 0.689 |
| TyG-BMI vs. HSI | 0.014 (0.010-0.019) | <0.001 | 0.024 (0.009-0.039) | 0.002 | 0.026 (0.007-0.045) | <0.001 |
| TyG-BMI vs. VAI | 0.109 (0.098-0.120) | <0.001 | 0.176 (0.152-0.200) | <0.001 | 0.164 (0.142-0.186) | <0.001 |
| FLI vs. TyG-BMI | 0.009 (0.006-0.013) | <0.001 | 0.008 (-0.006-0.022) | 0.251 | 0.012 (-0.007-0.031) | 0.206 |
| TyG-BMI vs. LAP | 0.016 (0.009-0.023) | <0.001 | 0.034 (0.016-0.052) | <0.001 | 0.037 (0.016-0.058) | 0.001 |
| TyG-BMI vs. ZJU | 0.005 (0.002-0.007) | <0.001 | 0.008 (-0.003-0.019) | 0.170 | 0.007 (-0.010-0.024) | 0.417 |
| FSI vs. TyG-BMI | 0.008 (0.004-0.013) | <0.001 | -0.002 (-0.018-0.014) | 0.810 | -0.006 (-0.026-0.014) | 0.559 |
| TyG-BMI vs. K-NAFLD | 0.010 (0.004-0.019) | 0.002 | 0.027 (0.007-0.047) | 0.007 | 0.031 (0.009-0.053) | 0.005 |
| TyG-WC vs. TyG-WtHR | 0.010 (0.006-0.013) | <0.001 | 0.011 (-0.003-0.025) | 0.137 | 0.019 (-0.000-0.038) | 0.053 |
| TyG-WC vs. HSI | 0.028 (0.021-0.034) | <0.001 | 0.039 (0.020-0.058) | <0.001 | 0.050 (0.028-0.072) | <0.001 |
| TyG-WC vs. VAI | 0.122 (0.112-0.132) | <0.001 | 0.192 (0.169-0.215) | <0.001 | 0.187 (0.165-0.209) | <0.001 |
| TyG-WC vs. FLI | 0.004 (0.000-0.007) | 0.027 | 0.007 (-0.007-0.021) | 0.313 | 0.011 (-0.008-0.030) | 0.251 |
| TyG-WC vs. LAP | 0.029 (0.024-0.034) | <0.001 | 0.049 (0.033-0.065) | <0.001 | 0.060 (0.040-0.080) | <0.001 |
| TyG-WC vs. ZJU | 0.018 (0.013-0.024) | <0.001 | 0.023 (0.006-0.040) | 0.010 | 0.030 (0.009-0.051) | 0.005 |
| TyG-WC vs. FSI | 0.004 (0.000-0.009) | 0.049 | 0.018 (0.001-0.035) | 0.042 | 0.029 (0.008-0.050) | 0.006 |
| TyG-WC vs. K-NAFLD | 0.025 (0.018-0.032) | <0.001 | 0.042 (0.023-0.061) | <0.001 | 0.054 (0.032-0.076) | <0.001 |
| TyG-WtHR vs. HSI | 0.018 (0.011-0.025) | <0.001 | 0.028 (0.009-0.047) | 0.004 | 0.030 (0.008-0.051) | 0.006 |
| TyG-WtHR vs. VAI | 0.112 (0.102-0.122) | <0.001 | 0.181 (0.158-0.204) | <0.001 | 0.167 (0.145-0.189) | <0.001 |
| FLI vs. TyG-WtHR | 0.006 (0.001-0.010) | 0.011 | 0.004 (-0.012-0.020) | 0.621 | 0.008 (-0.012-0.028) | 0.435 |
| TyG-WtHR vs. LAP | 0.036 (0.029-0.043) | <0.001 | 0.038 (0.022-0.054) | <0.001 | 0.041 (0.021-0.061) | <0.001 |
| TyG-WtHR vs. ZJU | 0.008 (0.003-0.014) | 0.001 | 0.012 (-0.005-0.029) | 0.156 | 0.011 (-0.010-0.031) | 0.289 |
| FSI vs. TyG-WtHR | 0.005 (0.001-0.010) | 0.048 | -0.007 (-0.024-0.010) | 0.419 | -0.009 (-0.030-0.012) | 0.390 |
| TyG-WtHR vs. K-NAFLD | 0.015 (0.009-0.022) | <0.001 | 0.031 (0.013-0.049) | 0.001 | 0.035 (0.014-0.056) | 0.001 |
| HSI vs. VAI | 0.094 (0.082-0.107) | <0.001 | 0.152 (0.126-0.178) | <0.001 | 0.137 (0.114-0.160) | <0.001 |
| FLI vs. HSI | 0.024 (0.018-0.029) | <0.001 | 0.032 (0.015-0.049) | <0.001 | 0.038 (0.017-0.059) | <0.001 |
| HSI vs. LAP | 0.001 (-0.008-0.011) | 0.747 | 0.010 (-0.012-0.032) | 0.370 | 0.011 (-0.012-0.034) | 0.343 |
| ZJU vs. HSI | 0.010 (0.006-0.013) | <0.001 | 0.016 (0.004-0.028) | 0.009 | 0.019 (0.002-0.036) | 0.030 |
| FSI vs. HSI | 0.023 (0.017-0.029) | <0.001 | 0.021 (0.003-0.039) | 0.023 | 0.021 (0.001-0.042) | 0.049 |
| K-NAFLD vs. HSI | 0.003 (-0.005-0.010) | 0.503 | 0.003 (-0.017-0.023) | 0.764 | 0.005 (-0.017-0.027) | 0.650 |
| FLI vs. VAI | 0.118 (0.107-0.129) | <0.001 | 0.185 (0.161-0.209) | <0.001 | 0.176 (0.154-0.198) | <0.001 |
| LAP vs. VAI | 0.093 (0.086-0.099) | <0.001 | 0.143 (0.125-0.161) | <0.001 | 0.126 (0.107-0.145) | <0.001 |
| ZJU vs. VAI | 0.104 (0.092-0.116) | <0.001 | 0.168 (0.143-0.193) | <0.001 | 0.157 (0.134-0.180) | <0.001 |
| FSI vs. VAI | 0.117 (0.107-0.128) | <0.001 | 0.174 (0.150-0.198) | <0.001 | 0.158 (0.136-0.180) | <0.001 |
| K-NAFLD vs. VAI | 0.097 (0.086-0.108) | <0.001 | 0.150 (0.125-0.175) | <0.001 | 0.133 (0.111-0.155) | <0.001 |
| FLI vs. LAP | 0.025 (0.019-0.031) | <0.001 | 0.042 (0.024-0.060) | <0.001 | 0.049 (0.028-0.070) | <0.001 |
| FLI vs. ZJU | 0.014 (0.009-0.019) | <0.001 | 0.016 (0.000-0.032) | 0.047 | 0.019 (-0.001-0.039) | 0.062 |
| FLI vs. FSI | 0.000 (-0.004-0.005) | 0.733 | 0.011 (-0.005-0.027) | 0.191 | 0.017 (-0.003-0.037) | 0.099 |
| FLI vs. K-NAFLD | 0.021 (0.014-0.028) | <0.001 | 0.035 (0.016-0.054) | <0.001 | 0.043 (0.022-0.064) | 0.099 |
| ZJU vs. LAP | 0.011 (0.003-0.019) | 0.007 | 0.026 (0.006-0.046) | 0.012 | 0.030 (0.008-0.052) | 0.008 |
| FSI vs. LAP | 0.024 (0.018-0.031) | <0.001 | 0.031 (0.012-0.050) | 0.001 | 0.032 (0.011-0.053) | 0.003 |
| K-NAFLD vs. LAP | 0.004 (-0.004-0.012) | 0.309 | 0.007 (-0.013-0.027) | 0.490 | 0.006 (-0.016-0.028) | 0.586 |
| FSI vs. ZJU | 0.013 (0.008-0.019) | <0.001 | 0.005 (-0.013-0.023) | 0.577 | 0.001 (-0.020-0.022) | 0.924 |
| ZJU vs. K-NAFLD | 0.007 (-0.000-0.014) | 0.057 | 0.019 (-0.000-0.038) | 0.050 | 0.024 (0.003-0.045) | 0.028 |
| FSI vs. K-NAFLD | 0.020 (0.014-0.027) | <0.001 | 0.024 (0.006-0.042) | 0.009 | 0.025 (0.004-0.050) | 0.019 |

**Supplement Table 2** Performance assessment of the noninvasive indices and models for the prediction of MAFLD in the Western China cohort

| Pairwise comparison | Difference AUC (95% CI) | *P* value | NRI (95% CI) | *P* value | IDI (95% CI) | *P* value |
| --- | --- | --- | --- | --- | --- | --- |
| METS-IR vs. TyG | 0.120 (0.109-0.131) | <0.001 | 0.215 (0.185-0.245) | <0.001 | 0.195 (0.167-0.223) | <0.001 |
| TyG-BMI vs. METS-IR | 0.007 (0.003-0.010) | <0.001 | 0.011 (-0.007-0.029) | 0.219 | 0.025 (-0.000-0.050) | 0.051 |
| METS-IR vs. TyG-WC | 0.023 (0.017-0.029) | <0.001 | 0.047 (0.026-0.068) | <0.001 | 0.053 (0.027-0.079) | <0.001 |
| METS-IR vs. TyG-WtHR | 0.030 (0.023-0.037) | <0.001 | 0.064 (0.043-0.085) | <0.001 | 0.072 (0.046-0.098) | <0.001 |
| METS-IR vs. HSI | 0.023 (0.015-0.032) | <0.001 | 0.057 (0.032-0.082) | <0.001 | 0.062 (0.034-0.090) | <0.001 |
| METS-IR vs. VAI | 0.123 (0.113-0.134) | <0.001 | 0.224 (0.197-0.251) | <0.001 | 0.208 (0.181-0.235) | <0.001 |
| METS-IR vs. FLI | 0.018 (0.012-0.024) | <0.001 | 0.037 (0.017-0.057) | <0.001 | 0.040 (0.014-0.066) | 0.002 |
| METS-IR vs. LAP | 0.042 (0.035-0.050) | <0.001 | 0.086 (0.063-0.109) | <0.001 | 0.088 (0.061-0.115) | <0.001 |
| ZJU vs. METS-IR | 0.003 (-0.002-0.009) | 0.238 | 0.003 (-0.017-0.023) | 0.774 | 0.006 (-0.020-0.032) | 0.656 |
| METS-IR vs. FSI | 0.024 (0.018-0.031) | <0.001 | 0.055 (0.034-0.076) | <0.001 | 0.063 (0.037-0.089) | <0.001 |
| METS-IR vs. K-NAFLD | 0.060 (0.050-0.070) | <0.001 | 0.110 (0.083-0.137) | <0.001 | 0.113 (0.085-0.141) | <0.001 |
| TyG-BMI vs. TyG | 0.127 (0.117-0.138) | <0.001 | 0.226 (0.178-0.252) | <0.001 | 0.220 (0.192-0.248) | <0.001 |
| TyG-WC vs. TyG | 0.097 (0.088-0.107) | <0.001 | 0.168 (0.140-0.196) | <0.001 | 0.142 (0.116-0.168) | <0.001 |
| TyG-WtHR vs. TyG | 0.091 (0.081-0.100) | <0.001 | 0.151 (0.123-0.179) | <0.001 | 0.123 (0.097-0.149) | <0.001 |
| HSI vs. TyG | 0.097 (0.083-0.112) | <0.001 | 0.158 (0.124-0.192) | <0.001 | 0.133 (0.104-0.162) | <0.001 |
| TyG vs. VAI | 0.003 (-0.003-0.009) | 0.340 | 0.009 (-0.012-0.030) | 0.409 | 0.013 (-0.009-0.035) | 0.239 |
| FLI vs. TyG | 0.103 (0.093-0.113) | <0.001 | 0.178 (0.150-0.206) | <0.001 | 0.155 (0.128-0.182) | <0.001 |
| LAP vs. TyG | 0.078 (0.071-0.085) | <0.001 | 0.130 (0.107-0.153) | <0.001 | 0.107 (0.083-0.131) | <0.001 |
| ZJU vs. TyG | 0.124 (0.112-0.135) | <0.001 | 0.218 (0.188-0.248) | <0.001 | 0.201 (0.173-0.229) | <0.001 |
| FSI vs. TyG | 0.096 (0.087-0.106) | <0.001 | 0.160 0.131-0.189) | <0.001 | 0.132 (0.106-0.158) | <0.001 |
| K-NAFLD vs. TyG | 0.060 (0.048-0.072) | <0.001 | 0.105 0.075-0.135) | <0.001 | 0.082 (0.055-0.109) | <0.001 |
| TyG-BMI vs. TyG-WC | 0.030 (0.024-0.035) | <0.001 | 0.058 (0.037-0.079) | <0.001 | 0.078 (0.052-0.104) | <0.001 |
| TyG-BMI vs. TyG-WtHR | 0.037 (0.031-0.042) | <0.001 | 0.076 (0.054-0.098) | <0.001 | 0.097 (0.071-0.123) | <0.001 |
| TyG-BMI vs. HSI | 0.030 (0.021-0.038) | <0.001 | 0.069 (0.044-0.094) | <0.001 | 0.087 (0.058-0.116) | <0.001 |
| TyG-BMI vs. VAI | 0.131 (0.119-0.141) | <0.001 | 0.236 (0.208-0.264) | <0.001 | 0.233 (0.205-0.261) | <0.001 |
| TyG-BMI vs. FLI | 0.024 (0.019-0.030) | <0.001 | 0.048 (0.028-0.068) | <0.001 | 0.065 (0.039-0.091) | <0.001 |
| TyG-BMI vs. LAP | 0.049 (0.043-0.056) | <0.001 | 0.097 (0.075-0.119) | <0.001 | 0.113 (0.086-0.140) | <0.001 |
| TyG-BMI vs. ZJU | 0.003 (-0.001-0.008) | 0.145 | 0.009 (-0.009-0.028) | 0.346 | 0.019 (-0.006-0.044) | 0.143 |
| TyG-BMI vs. FSI | 0.031 (0.025-0.037) | <0.001 | 0.067 (0.047-0.087) | <0.001 | 0.088 (0.062-0.114) | <0.001 |
| TyG-BMI vs. K-NAFLD | 0.067 (0.058-0.076) | <0.001 | 0.122 (0.096-0.148) | <0.001 | 0.138 (0.110-0.166) | <0.001 |
| TyG-WC vs. TyG-WtHR | 0.007 (0.002-0.011) | 0.002 | 0.018 (0.000-0.036) | 0.047 | 0.019 (-0.004-0.042) | 0.108 |
| TyG-WC vs. HSI | 0.000 (-0.010-0.010) | 0.981 | 0.011 (-0.017-0.039) | 0.433 | 0.009 (-0.020-0.038) | 0.544 |
| TyG-WC vs. VAI | 0.100 (0.090-0.111) | <0.001 | 0.177 (0.150-0.204) | <0.001 | 0.155 (0.129-0.181) | <0.001 |
| FLI vs. TyG-WC | 0.005 (0.001-0.010) | 0.015 | 0.010 (-0.007-0.027) | 0.313 | 0.013 (-0.011-0.037) | 0.279 |
| TyG-WC vs. LAP | 0.019 (0.015-0.024) | <0.001 | 0.039 (0.019-0.059) | <0.001 | 0.035 (0.011-0.059) | 0.004 |
| ZJU vs. TyG-WC | 0.026 (0.019-0.034) | <0.001 | 0.050 (0.026-0.074) | <0.001 | 0.058 (0.030-0.086) | <0.001 |
| TyG-WC vs. FSI | 0.001 (-0.005-0.007) | 0.754 | 0.009 (-0.011-0.029) | 0.386 | 0.010 (-0.015-0.035) | 0.431 |
| TyG-WC vs. K-NAFLD | 0.037 (0.028-0.047) | <0.001 | 0.063 (0.037-0.089) | <0.001 | 0.061 (0.034-0.088) | <0.001 |
| HSI vs. TyG-WtHR | 0.007 (-0.004-0.017) | 0.211 | 0.007 (-0.020-0.034) | 0.613 | 0.010 (-0.018-0.038) | 0.491 |
| TyG-WtHR vs. VAI | 0.094 (0.083-0.104) | <0.001 | 0.160 (0.133-0.187) | <0.001 | 0.136 (0.111-0.161) | <0.001 |
| FLI vs. TyG-WtHR | 0.012 (0.006-0.018) | <0.001 | 0.027 (0.007-0.047) | 0.009 | 0.032 (0.007-0.057) | 0.012 |
| TyG-WtHR vs. LAP | 0.013 (0.008-0.018) | <0.001 | 0.021 (0.001-0.041) | 0.042 | 0.016 (-0.008-0.040) | 0.191 |
| ZJU vs. TyG-WtHR | 0.033 (0.026-0.040) | <0.001 | 0.067 (0.044-0.090) | <0.001 | 0.078 (0.051-0.105) | <0.001 |
| FSI vs. TyG-WtHR | 0.006 (-0.001-0.013) | 0.091 | 0.009 (-0.010-0.028) | 0.359 | 0.009 (-0.015-0.033) | 0.461 |
| TyG-WtHR vs. K-NAFLD | 0.030 (0.022-0.039) | <0.001 | 0.046 (0.021-0.071) | <0.001 | 0.041 (0.015-0.067) | 0.002 |
| HSI vs. VAI | 0.100 (0.086-0.115) | <0.001 | 0.167 (0.135-0.199) | <0.001 | 0.146 (0.117-0.175) | <0.001 |
| FLI vs. HSI | 0.006 (-0.015-0.004) | 0.237 | 0.020 (-0.006-0.046) | 0.129 | 0.022 (-0.006-0.050) | 0.129 |
| HSI vs. LAP | 0.020 (0.008-0.030) | 0.747 | 0.028 (-0.001-0.057) | 0.056 | 0.026 (-0.003-0.055) | 0.082 |
| ZJU vs. HSI | 0.027 (0.021-0.032) | <0.001 | 0.060 (0.041-0.079) | <0.001 | 0.067 (0.042-0.092) | <0.001 |
| HSI vs. FSI | 0.001 (-0.007-0.009) | 0.829 | -0.002 (-0.025-0.021) | 0.867 | 0.074 (-0.025-0.027) | 0.941 |
| HSI vs. K-NAFLD | 0.037 (0.028-0.046) | <0.001 | 0.053 (0.028-0.078) | <0.001 | 0.052 (0.025-0.079) | <0.001 |
| FLI vs. VAI | 0.106 (0.095-0.117) | <0.001 | 0.187 (0.160-0.214) | <0.001 | 0.168 (0.141-0.195) | <0.001 |
| LAP vs. VAI | 0.081 (0.074-0.088) | <0.001 | 0.139 (0.117-0.161) | <0.001 | 0.120 (0.097-0.143) | <0.001 |
| ZJU vs. VAI | 0.127 (0.115-0.139) | <0.001 | 0.227 (0.198-0.256) | <0.001 | 0.214 (0.186-0.242) | <0.001 |
| FSI vs. VAI | 0.099 (0.089-0.110) | <0.001 | 0.169 (0.142-0.196) | <0.001 | 0.145 (0.119-0.171) | <0.001 |
| K-NAFLD vs. VAI | 0.063 (0.051-0.076) | <0.001 | 0.114 (0.084-0.144) | <0.001 | 0.095 (0.068-0.122) | <0.001 |
| FLI vs. LAP | 0.025 (0.019-0.030) | <0.001 | 0.049 (0.028-0.070) | <0.001 | 0.048 (0.023-0.073) | <0.001 |
| ZJU vs. FLI | 0.021 (0.014-0.028) | <0.001 | 0.040 (0.017-0.063) | 0.001 | 0.045 (0.017-0.073) | 0.001 |
| FLI vs. FSI | 0.006 (0.001-0.012) | 0.027 | 0.011 (-0.002-0.038) | 0.081 | 0.023 (-0.002-0.048) | 0.070 |
| FLI vs. K-NAFLD | 0.043 (0.034-0.051) | <0.001 | 0.073 (0.048-0.098) | <0.001 | 0.074 (0.047-0.101) | <0.001 |
| ZJU vs. LAP | 0.046 (0.038-0.054) | <0.001 | 0.088 (0.062-0.113) | <0.001 | 0.094 (0.066-0.122) | <0.001 |
| FSI vs. LAP | 0.018 (0.012-0.025) | <0.001 | 0.030 (0.008-0.052) | 0.008 | 0.025 (-0.000-0.050) | 0.053 |
| LAP vs. K-NAFLD | 0.018 (0.008-0.027) | <0.001 | 0.025 (-0.000-0.050) | 0.052 | 0.025 (-0.002-0.052) | 0.067 |
| ZJU vs. FSI | 0.027 (0.021-0.034) | <0.001 | 0.058 (0.037-0.079) | <0.001 | 0.069 (0.043-0.095) | <0.001 |
| ZJU vs. K-NAFLD | 0.064 (0.056-0.071) | <0.001 | 0.113 (0.090-0.136) | <0.001 | 0.119 (0.093-0.145) | <0.001 |
| FSI vs. K-NAFLD | 0.036 (0.029-0.044) | <0.001 | 0.055 (0.032-0.078) | <0.001 | 0.050 (0.024-0.076) | <0.001 |

**Supplement Table 3** Subgroup analysis of METS-IR for predicting MAFLD risk in the NHANES cohort

| Subgroup | AUC  (95% CI) | SEN  (95% CI) | SPE  (95% CI) | PPV  (95% CI) | NPV  (95% CI) | Cutoff value |
| --- | --- | --- | --- | --- | --- | --- |
| Sex | | | | | | |
| Female | 0.837  (0.825-0.850) | 0.797  (0.778-0.816) | 0.719  (0.699-0.739) | 0.713  (0.693-0.733) | 0.802  (0.783-0.820) | 41.056 |
| Male | 0.856  (0.844-0.868) | 0.810  (0.793-0.827) | 0.744  (0.723-0.765) | 0.793  (0.775-0.810) | 0.764  (0.743-0.785) | 41.483 |
| Race |  |  |  |  |  |  |
| Black | 0.826  (0.808-0.845) | 0.792  (0.765-0.820) | 0.706  (0.678-0.734) | 0.685  (0.655-0.714) | 0.808  (0.783-0.834) | 42.763 |
| White | 0.869  (0.855-0.882) | 0.820  (0.799-0.840) | 0.759  (0.735-0.783) | 0.789  (0.768-0.810) | 0.793  (0.770-0.816) | 40.796 |
| Hispanic | 0.824  (0.795-0.853) | 0.824  (0.787-0.861) | 0.669  (0.620-0.717) | 0.737  (0.697-0.778) | 0.771  (0.724-0.817) | 41.677 |
| Asian | 0.859  (0.835-0.883) | 0.873  (0.839-0.907) | 0.712  (0.672-0.751) | 0.690  (0.649-0.732) | 0.884  (0.853-0.915) | 36.630 |
| Mexican | 0.842  (0.815-0.869) | 0.753  (0.719-0.788) | 0.789  (0.745-0.833) | 0.865  (0.836-0.895) | 0.640  (0.593-0.686) | 43.757 |
| Other | 0.841  (0.800-0.882) | 0.860  (0.810-0.911) | 0.722  (0.657-0.788) | 0.755  (0.696-0.814) | 0.839  (0.781-0.897) | 41.158 |
| Age | | | | | | |
| <60 | 0.868  (0.858-0.878) | 0.842  (0.827-0.856) | 0.741  (0.724-0.757) | 0.743  (0.726-0.759) | 0.840  (0.825-0.855) | 41.307 |
| ≥60 | 0.800  (0.783-0.818) | 0.752  (0.730-0.775) | 0.704  (0.676-0.732) | 0.777  (0.755-0.799) | 0.675  (0.647-0.703) | 41.099 |
| Overweight | | | | | | |
| No | 0.803  (0.768-0.837) | 0.668  (0.603-0.734) | 0.812  (0.793-0.830) | 0.287  (0.245-0.328) | 0.956  (0.945-0.966) | 33.698 |
| Yes | 0.749  (0.736-0.762) | 0.734  (0.719-0.748) | 0.632  (0.610-0.653) | 0.789  (0.775-0.803) | 0.558  (0.537-0.579) | 44.048 |
| Hypertension | | | | | | |
| No | 0.860  (0.850-0.871) | 0.807  (0.790-0.825) | 0.758  (0.742-0.774) | 0.713  (0.695-0.732) | 0.841  (0.826-0.855) | 40.858 |
| Yes | 0.798  (0.780-0.815) | 0.729  (0.708-0.750) | 0.723  (0.694-0.751) | 0.828  (0.809-0.847) | 0.593  (0.565-0.621) | 44.035 |
| Diabetes | | | | | | |
| No | 0.843  (0.833-0.853) | 0.812  (0.797-0.827) | 0.717  (0.701-0.732) | 0.695  (0.678-0.711) | 0.827  (0.814-0.841) | 40.101 |
| Yes | 0.774  (0.744-0.803) | 0.782  (0.758-0.806) | 0.634  (0.581-0.686) | 0.879  (0.859-0.900) | 0.460  (0.414-0.506) | 44.120 |

Note: Black means non-Hispanic Black, White means non-Hispanic White, Hispanic means other Hispanic, Asian means non-Hispanic Asian, Mexican means Mexican American, and Other means other races.

**Supplement Table 4** Subgroup analysis of TyG for predicting MAFLD risk in the NHANES cohort

| Subgroup | AUC  (95% CI) | SEN  (95% CI) | SPE  (95% CI) | PPV  (95% CI) | NPV  (95% CI) | Cutoff value |
| --- | --- | --- | --- | --- | --- | --- |
| Sex | | | | | | |
| Female | 0.750  (0.735-0.765) | 0.733  (0.712-0.754) | 0.640  (0.619-0.661) | 0.641  (0.620-0.662) | 0.732  (0.712-0.753) | 8.444 |
| Male | 0.738  (0.722-0.754) | 0.754  (0.736-0.773) | 0.598  (0.574-0.621) | 0.694  (0.675-0.713) | 0.668  (0.644-0.692) | 8.497 |
| Race |  |  |  |  |  |  |
| Black | 0.718  (0.695-0.741) | 0.658  (0.626-0.691) | 0.677  (0.648-0.706) | 0.621  (0.589-0.654) | 0.711  (0.683-0.739) | 8.368 |
| White | 0.764  (0.746-0.782) | 0.772  (0.750-0.794) | 0.617  (0.590-0.644) | 0.689  (0.666-0.712) | 0.711  (0.684-0.738) | 8.497 |
| Hispanic | 0.737  (0.702-0.772) | 0.623  (0.577-0.670) | 0.751  (0.707-0.796) | 0.739  (0.693-0.785) | 0.638  (0.593-0.684) | 8.796 |
| Asian | 0.782  (0.753-0.812) | 0.725  (0.680-0.770) | 0.706  (0.666-0.745) | 0.645  (0.599-0.690) | 0.777  (0.739-0.815) | 8.715 |
| Mexican | 0.731  (0.698-0.765) | 0.753  (0.719-0.788) | 0.607  (0.555-0.660) | 0.775  (0.741-0.809) | 0.578  (0.526-0.629) | 8.527 |
| Other | 0.732  (0.680-0.784) | 0.693  (0.625-0.760) | 0.683  (0.615-0.751) | 0.685  (0.617-0.753) | 0.691  (0.623-0.759) | 8.545 |
| Age | | | | | | |
| <60 | 0.758  (0.745-0.771) | 0.703  (0.684-0.721) | 0.688  (0.670-0.705) | 0.667  (0.648-0.686) | 0.722  (0.704-0.740) | 8.497 |
| ≥60 | 0.708  (0.687-0.728) | 0.518  (0.492-0.544) | 0.783  (0.758-0.808) | 0.766  (0.739-0.792) | 0.543  (0.518-0.568) | 8.875 |
| Overweight | | | | | | |
| No | 0.829  (0.799-0.859) | 0.618  (0.551-0.686) | 0.882  (0.867-0.897) | 0.372  (0.320-0.424) | 0.953  (0.943-0.963) | 8.865 |
| Yes | 0.700  (0.686-0.715) | 0.725  (0.710-0.740) | 0.571  (0.549-0.593) | 0.760  (0.746-0.775) | 0.525  (0.504-0.547) | 8.497 |
| Hypertension | | | | | | |
| No | 0.752  (0.739-0.766) | 0.703  (0.683-0.723) | 0.679  (0.662-0.697) | 0.621  (0.601-0.641) | 0.754  (0.737-0.771) | 8.497 |
| Yes | 0.701  (0.681-0.722) | 0.679  (0.657-0.701) | 0.613  (0.582-0.644) | 0.762  (0.741-0.784) | 0.511  (0.482-0.540) | 8.639 |
| Diabetes | | | | | | |
| No | 0.724  (0.711-0.737) | 0.764  (0.747-0.780) | 0.575  (0.558-0.591) | 0.588  (0.571-0.604) | 0.754  (0.737-0.771) | 8.368 |
| Yes | 0.682  (0.649-0.715) | 0.737  (0.711-0.763) | 0.538  (0.484-0.593) | 0.845  (0.822-0.868) | 0.375  (0.331-0.419) | 8.805 |

Note: Black means non-Hispanic Black, White means non-Hispanic White, Hispanic means other Hispanic, Asian means non-Hispanic Asian, Mexican means Mexican American, and Other means other races.

**Supplement Table 5** Subgroup analysis of TyG-BMI for predicting MAFLD risk in the NHANES cohort

| Subgroup | AUC  (95% CI) | SEN  (95% CI) | SPE  (95% CI) | PPV  (95% CI) | NPV  (95% CI) | Cutoff value |
| --- | --- | --- | --- | --- | --- | --- |
| Sex | | | | | | |
| Female | 0.842  (0.830-0.855) | 0.825  (0.807-0.843) | 0.699  (0.679-0.720) | 0.706  (0.687-0.726) | 0.820  (0.802-0.838) | 241.013 |
| Male | 0.865  (0.853-0.876) | 0.809  (0.792-0.827) | 0.765  (0.745-0.786) | 0.807  (0.789-0.824) | 0.769  (0.748-0.789) | 240.215 |
| Race |  |  |  |  |  |  |
| Black | 0.834  (0.816-0.852) | 0.779  (0.751-0.807) | 0.735  (0.708-0.762) | 0.703  (0.674-0.733) | 0.805  (0.780-0.830) | 254.093 |
| White | 0.873  (0.860-0.887) | 0.823  (0.802-0.843) | 0.755  (0.731-0.779) | 0.787  (0.765-0.808) | 0.795  (0.771-0.818) | 240.222 |
| Hispanic | 0.821  (0.792-0.851) | 0.836  (0.800-0.872) | 0.671  (0.623-0.720) | 0.742  (0.702-0.782) | 0.784  (0.738-0.830) | 243.412 |
| Asian | 0.873  (0.850-0.895) | 0.817  (0.779-0.856) | 0.782  (0.746-0.817) | 0.734  (0.692-0.776) | 0.853  (0.821-0.885) | 224.313 |
| Mexican | 0.844  (0.817-0.871) | 0.826  (0.795-0.856) | 0.725  (0.677-0.773) | 0.844  (0.814-0.873) | 0.698  (0.649-0.746) | 245.582 |
| Other | 0.835  (0.793-0.876) | 0.855  (0.803-0.906) | 0.700  (0.633-0.767) | 0.739  (0.679-0.799) | 0.829  (0.769-0.889) | 237.740 |
| Age | | | | | | |
| <60 | 0.871  (0.862-0.881) | 0.843  (0.828-0.858) | 0.748  (0.732-0.765) | 0.749  (0.732-0.765) | 0.842  (0.828-0.857) | 240.240 |
| ≥60 | 0.802  (0.784-0.819) | 0.747  (0.724-0.769) | 0.709  (0.681-0.736) | 0.778  (0.756-0.800) | 0.671  (0.644-0.699) | 245.031 |
| Overweight | | | | | | |
| No | 0.839  (0.811-0.866) | 0.739  (0.678-0.800) | 0.787  (0.767-0.806) | 0.282  (0.243-0.320) | 0.964  (0.954-0.973) | 199.023 |
| Yes | 0.753  (0.740-0.767) | 0.692  (0.677-0.707) | 0.686  (0.665-0.706) | 0.805  (0.791-0.819) | 0.542  (0.522-0.562) | 263.307 |
| Hypertension | | | | | | |
| No | 0.866  (0.856-0.876) | 0.886  (0.872-0.900) | 0.692  (0.675-0.710) | 0.682  (0.664-0.700) | 0.890  (0.877-0.904) | 227.931 |
| Yes | 0.798  (0.780-0.815) | 0.725  (0.704-0.746) | 0.730  (0.702-0.758) | 0.831  (0.812-0.850) | 0.592  (0.564-0.621) | 258.723 |
| Diabetes | | | | | | |
| No | 0.849  (0.839-0.858) | 0.788  (0.772-0.803) | 0.751  (0.736-0.766) | 0.715  (0.699-0.732) | 0.817  (0.803-0.830) | 240.207 |
| Yes | 0.775  (0.746-0.805) | 0.771  (0.746-0.796) | 0.646  (0.594-0.698) | 0.882  (0.861-0.902) | 0.453  (0.407-0.498) | 259.111 |

Note: Black means non-Hispanic Black, White means non-Hispanic White, Hispanic means other Hispanic, Asian means non-Hispanic Asian, Mexican means Mexican American, and Other means other races.

**Supplement Table 6** Subgroup analysis of TyG-WC for predicting MAFLD risk in the NHANES cohort

| Subgroup | AUC  (95% CI) | SEN  (95% CI) | SPE  (95% CI) | PPV  (95% CI) | NPV  (95% CI) | Cutoff value |
| --- | --- | --- | --- | --- | --- | --- |
| Sex | | | | | | |
| Female | 0.854  (0.843-0.866) | 0.875  (0.859-0.890) | 0.671  (0.651-0.692) | 0.700  (0.681-0.719) | 0.860  (0.842-0.877) | 797.961 |
| Male | 0.870  (0.859-0.882) | 0.847  (0.831-0.863) | 0.733  (0.711-0.754) | 0.793  (0.776-0.810) | 0.798  (0.778-0.818) | 846.383 |
| Race |  |  |  |  |  |  |
| Black | 0.849  (0.832-0.866) | 0.797  (0.770-0.824) | 0.745  (0.718-0.772) | 0.716  (0.687-0.745) | 0.820  (0.795-0.845) | 855.415 |
| White | 0.885  (0.873-0.898) | 0.869  (0.851-0.887) | 0.737  (0.712-0.761) | 0.784  (0.763-0.805) | 0.836  (0.814-0.858) | 839.965 |
| Hispanic | 0.833  (0.806-0.861) | 0.780  (0.740-0.820) | 0.713  (0.666-0.759) | 0.754  (0.713-0.795) | 0.741  (0.695-0.787) | 849.271 |
| Asian | 0.869  (0.846-0.891) | 0.810  (0.770-0.849) | 0.776  (0.740-0.812) | 0.727  (0.684-0.769) | 0.847  (0.814-0.879) | 800.319 |
| Mexican | 0.854  (0.828-0.880) | 0.723  (0.687-0.759) | 0.831  (0.790-0.871) | 0.885  (0.857-0.913) | 0.625  (0.580-0.670) | 878.284 |
| Other | 0.860  (0.822-0.897) | 0.849  (0.797-0.902) | 0.728  (0.663-0.793) | 0.756  (0.697-0.816) | 0.829  (0.770-0.888) | 842.217 |
| Age | | | | | | |
| <60 | 0.881  (0.872-0.890) | 0.857  (0.842-0.871) | 0.747  (0.730-0.763) | 0.751  (0.734-0.767) | 0.854  (0.840-0.869) | 816.374 |
| ≥60 | 0.819  (0.802-0.835) | 0.765  (0.743-0.787) | 0.711  (0.683-0.738) | 0.783  (0.762-0.805) | 0.688  (0.661-0.716) | 870.642 |
| Overweight | | | | | | |
| No | 0.870  (0.846-0.893) | 0.869  (0.823-0.916) | 0.738  (0.718-0.759) | 0.273  (0.239-0.308) | 0.980  (0.973-0.988) | 722.364 |
| Yes | 0.781  (0.768-0.793) | 0.667  (0.652-0.683) | 0.747  (0.727-0.766) | 0.832  (0.818-0.845) | 0.545  (0.525-0.564) | 908.908 |
| Hypertension | | | | | | |
| No | 0.875  (0.866-0.885) | 0.846  (0.830-0.861) | 0.751  (0.735-0.768) | 0.717  (0.699-0.735) | 0.867  (0.853-0.881) | 816.374 |
| Yes | 0.814  (0.798-0.831) | 0.706  (0.684-0.727) | 0.772  (0.745-0.799) | 0.850  (0.832-0.868) | 0.589  (0.562-0.617) | 911.493 |
| Diabetes | | | | | | |
| No | 0.861  (0.851-0.870) | 0.845  (0.831-0.858) | 0.720  (0.705-0.735) | 0.705  (0.689-0.721) | 0.854  (0.841-0.867) | 816.374 |
| Yes | 0.782  (0.754-0.811) | 0.795  (0.772-0.819) | 0.643  (0.591-0.695) | 0.884  (0.864-0.904) | 0.479  (0.432-0.526) | 911.897 |

Note: Black means non-Hispanic Black, White means non-Hispanic White, Hispanic means other Hispanic, Asian means non-Hispanic Asian, Mexican means Mexican American, and Other means other races.

**Supplement Table 7** Subgroup analysis of TyG-WtHR for predicting MAFLD risk in the NHANES cohort

| Subgroup | AUC  (95% CI) | SEN  (95% CI) | SPE  (95% CI) | PPV  (95% CI) | NPV  (95% CI) | Cutoff value |
| --- | --- | --- | --- | --- | --- | --- |
| Sex | | | | | | |
| Female | 0.853  (0.841-0.864) | 0.851  (0.834-0.868) | 0.705  (0.685-0.725) | 0.717  (0.697-0.736) | 0.844  (0.826-0.861) | 5.086 |
| Male | 0.867  (0.856-0.879) | 0.813  (0.796-0.830) | 0.759  (0.739-0.780) | 0.803  (0.786-0.821) | 0.771  (0.750-0.791) | 4.962 |
| Race |  |  |  |  |  |  |
| Black | 0.836  (0.818-0.853) | 0.801  (0.774-0.828) | 0.732  (0.705-0.760) | 0.707  (0.678-0.736) | 0.820  (0.795-0.845) | 5.067 |
| White | 0.871  (0.858-0.884) | 0.824  (0.804-0.844) | 0.744  (0.720-0.768) | 0.780  (0.759-0.801) | 0.794  (0.770-0.817) | 5.060 |
| Hispanic | 0.826  (0.796-0.855) | 0.822  (0.784-0.859) | 0.702  (0.655-0.749) | 0.757  (0.717-0.797) | 0.777  (0.732-0.822) | 5.146 |
| Asian | 0.869  (0.846-0.892) | 0.865  (0.831-0.900) | 0.727  (0.689-0.766) | 0.700  (0.659-0.742) | 0.880  (0.849-0.911) | 4.770 |
| Mexican | 0.835  (0.808-0.863) | 0.782  (0.749-0.815) | 0.749  (0.703-0.796) | 0.849  (0.819-0.879) | 0.656  (0.608-0.704) | 5.214 |
| Other | 0.852  (0.813-0.891) | 0.872  (0.822-0.921) | 0.700  (0.633-0.767) | 0.743  (0.684-0.802) | 0.846  (0.788-0.904) | 4.887 |
| Age | | | | | | |
| <60 | 0.875  (0.866-0.885) | 0.823  (0.807-0.839) | 0.775  (0.759-0.791) | 0.765  (0.748-0.781) | 0.831  (0.816-0.846) | 4.981 |
| ≥60 | 0.800  (0.783-0.818) | 0.731  (0.708-0.754) | 0.714  (0.687-0.742) | 0.778  (0.756-0.800) | 0.660  (0.632-0.687) | 5.352 |
| Overweight | | | | | | |
| No | 0.874  (0.850-0.897) | 0.809  (0.754-0.864) | 0.784  (0.764-0.803) | 0.298  (0.259-0.336) | 0.973  (0.965-0.982) | 4.445 |
| Yes | 0.763  (0.750-0.777) | 0.702  (0.687-0.717) | 0.687  (0.666-0.707) | 0.808  (0.794-0.822) | 0.551  (0.531-0.571) | 5.376 |
| Hypertension | | | | | | |
| No | 0.869  (0.859-0.879) | 0.813  (0.796-0.830) | 0.770  (0.755-0.786) | 0.726  (0.707-0.744) | 0.847  (0.833-0.861) | 4.962 |
| Yes | 0.796  (0.778-0.813) | 0.743  (0.722-0.763) | 0.699  (0.670-0.729) | 0.819  (0.800-0.838) | 0.598  (0.569-0.627) | 5.375 |
| Diabetes | | | | | | |
| No | 0.850  (0.840-0.859) | 0.804  (0.789-0.819) | 0.740  (0.725-0.755) | 0.711  (0.694-0.727) | 0.826  (0.812-0.840) | 4.981 |
| Yes | 0.774  (0.745-0.803) | 0.686  (0.658-0.713) | 0.735  (0.687-0.783) | 0.898  (0.878-0.919) | 0.406  (0.367-0.446) | 5.797 |

Note: Black means non-Hispanic Black, White means non-Hispanic White, Hispanic means other Hispanic, Asian means non-Hispanic Asian, Mexican means Mexican American, and Other means other races.

**Supplement Table 8** Subgroup analysis of HSI for predicting MAFLD risk in the NHANES cohort

| Subgroup | AUC  (95% CI) | SEN  (95% CI) | SPE  (95% CI) | PPV  (95% CI) | NPV  (95% CI) | Cutoff value |
| --- | --- | --- | --- | --- | --- | --- |
| Sex | | | | | | |
| Female | 0.829  (0.816-0.841) | 0.771  (0.752-0.791) | 0.734  (0.715-0.753) | 0.718  (0.697-0.738) | 0.786  (0.767-0.804) | 38.505 |
| Male | 0.856  (0.844-0.868) | 0.776  (0.758-0.794) | 0.781  (0.761-0.801) | 0.811  (0.793-0.828) | 0.742  (0.722-0.763) | 37.019 |
| Race |  |  |  |  |  |  |
| Black | 0.827  (0.809-0.846) | 0.771  (0.742-0.799) | 0.747  (0.721-0.774) | 0.710  (0.681-0.740) | 0.802  (0.776-0.827) | 39.471 |
| White | 0.851  (0.837-0.865) | 0.751  (0.728-0.774) | 0.789  (0.767-0.812) | 0.797  (0.775-0.819) | 0.742  (0.718-0.766) | 37.910 |
| Hispanic | 0.809  (0.779-0.840) | 0.792  (0.753-0.831) | 0.682  (0.634-0.730) | 0.738  (0.697-0.779) | 0.744  (0.697-0.791) | 37.768 |
| Asian | 0.855  (0.830-0.880) | 0.794  (0.753-0.834) | 0.778  (0.742-0.814) | 0.725  (0.682-0.768) | 0.836  (0.803-0.870) | 34.783 |
| Mexican | 0.838  (0.811-0.865) | 0.790  (0.758-0.823) | 0.749  (0.703-0.769) | 0.850  (0.820-0.880) | 0.665  (0.617-0.713) | 38.318 |
| Other | 0.824  (0.781-0.867) | 0.860  (0.810-0.911) | 0.689  (0.621-0.757) | 0.733  (0.674-0.793) | 0.832  (0.722-0.892) | 36.519 |
| Age | | | | | | |
| <60 | 0.863  (0.853-0.873) | 0.842  (0.827-0.857) | 0.730  (0.713-0.747) | 0.735  (0.719-0.752) | 0.838  (0.823-0.854) | 37.224 |
| ≥60 | 0.785  (0.767-0.803) | 0.726  (0.703-0.749) | 0.712  (0.684-0.739) | 0.775  (0.753-0.797) | 0.655  (0.627-0.682) | 37.128 |
| Overweight | | | | | | |
| No | 0.750  (0.716-0.783) | 0.754  (0.694-0.814) | 0.622  (0.599-0.645) | 0.184  (0.158-0.211) | 0.957  (0.945-0.969) | 30.606 |
| Yes | 0.738  (0.725-0.752) | 0.711  (0.696-0.726) | 0.653  (0.632-0.675) | 0.794  (0.780-0.808) | 0.546  (0.526-0.567) | 39.514 |
| Hypertension | | | | | | |
| No | 0.852  (0.841-0.862) | 0.838  (0.822-0.854) | 0.714  (0.697-0.731) | 0.686  (0.668-0.705) | 0.855  (0.841-0.870) | 36.163 |
| Yes | 0.785  (0.767-0.803) | 0.710  (0.689-0.732) | 0.739  (0.712-0.767) | 0.833  (0.814-0.852) | 0.583  (0.555-0.611) | 39.461 |
| Diabetes | | | | | | |
| No | 0.830  (0.820-0.840) | 0.798  (0.783-0.813) | 0.707  (0.692-0.723) | 0.684  (0.668-0.700) | 0.815  (0.801-0.829) | 36.410 |
| Yes | 0.767  (0.737-0.796) | 0.788  (0.764-0.812) | 0.618  (0.566-0.671) | 0.876  (0.855-0.896) | 0.461  (0.414-0.508) | 39.474 |

Note: Black means non-Hispanic Black, White means non-Hispanic White, Hispanic means other Hispanic, Asian means non-Hispanic Asian, Mexican means Mexican American, and Other means other races.

**Supplement Table 9** Subgroup analysis of VAI for predicting MAFLD risk in the NHANES cohort

| Subgroup | AUC  (95% CI) | SEN  (95% CI) | SPE  (95% CI) | PPV  (95% CI) | NPV  (95% CI) | Cutoff value |
| --- | --- | --- | --- | --- | --- | --- |
| Sex | | | | | | |
| Female | 0.752  (0.736-0.767) | 0.752  (0.732-0.772) | 0.634  (0.613-0.655) | 0.643  (0.622-0.664) | 0.745  (0.724-0.765) | 1.499 |
| Male | 0.740  (0.724-0.756) | 0.666  (0.646-0.687) | 0.703  (0.681-0.725) | 0.731  (0.710-0.751) | 0.635  (0.613-0.657) | 1.526 |
| Race |  |  |  |  |  |  |
| Black | 0.720  (0.697-0.743) | 0.628  (0.595-0.661) | 0.712  (0.684-0.740) | 0.637  (0.604-0.670) | 0.704  (0.676-0.732) | 1.304 |
| White | 0.752  (0.734-0.771) | 0.690  (0.666-0.715) | 0.689  (0.663-0.715) | 0.710  (0.685-0.734) | 0.669  (0.643-0.695) | 1.654 |
| Hispanic | 0.714  (0.678-0.750) | 0.699  (0.655-0.744) | 0.619  (0.569-0.669) | 0.675  (0.630-0.719) | 0.646  (0.595-0.696) | 1.669 |
| Asian | 0.769  (0.739-0.800) | 0.780  (0.739-0.822) | 0.663  (0.622-0.704) | 0.630  (0.587-0.674) | 0.804  (0.766-0.842) | 1.635 |
| Mexican | 0.731  (0.696-0.765) | 0.725  (0.689-0.761) | 0.665  (0.614-0.716) | 0.796  (0.762-0.830) | 0.573  (0.523-0.622) | 1.618 |
| Other | 0.731  (0.679-0.783) | 0.799  (0.740-0.858) | 0.600  (0.528-0.672) | 0.665  (0.602-0.728) | 0.750  (0.679-0.821) | 1.265 |
| Age | | | | | | |
| <60 | 0.758  (0.744-0.771) | 0.718  (0.699-0.736) | 0.677  (0.659-0.695) | 0.664  (0.645-0.683) | 0.729  (0.711-0.747) | 1.494 |
| ≥60 | 0.701  (0.681-0.722) | 0.666  (0.642-0.691) | 0.655  (0.626-0.684) | 0.726  (0.702-0.750) | 0.589  (0.561-0.618) | 1.612 |
| Overweight | | | | | | |
| No | 0.789  (0.752-0.826) | 0.683  (0.619-0.748) | 0.788  (0.769-0.807) | 0.267  (0.229-0.306) | 0.956  (0.946-0.967) | 1.577 |
| Yes | 0.683  (0.668-0.698) | 0.645  (0.630-0.661) | 0.633  (0.611-0.655) | 0.768  (0.752-0.783) | 0.488  (0.468-0.507) | 1.673 |
| Hypertension | | | | | | |
| No | 0.755  (0.741-0.769) | 0.760  (0.741-0.778) | 0.631  (0.612-0.649) | 0.605  (0.586-0.624) | 0.779  (0.761-0.796) | 1.339 |
| Yes | 0.695  (0.674-0.716) | 0.668  (0.646-0.691) | 0.636  (0.605-0.667) | 0.771  (0.749-0.792) | 0.512  (0.483-0.540) | 1.657 |
| Diabetes | | | | | | |
| No | 0.729  (0.716-0.741) | 0.728  (0.711-0.745) | 0.621  (0.604-0.637) | 0.604  (0.587-0.621) | 0.742  (0.726-0.758) | 1.350 |
| Yes | 0.701  (0.668-0.735) | 0.650  (0.622-0.679) | 0.683  (0.632-0.734) | 0.875  (0.853-0.898) | 0.364  (0.326-0.402) | 1.975 |

Note: Black means non-Hispanic Black, White means non-Hispanic White, Hispanic means other Hispanic, Asian means non-Hispanic Asian, Mexican means Mexican American, and Other means other races.

**Supplement Table 10** Subgroup analysis of FLI for predicting MAFLD risk in the NHANES cohort

| Subgroup | AUC  (95% CI) | SEN  (95% CI) | SPE  (95% CI) | PPV  (95% CI) | NPV  (95% CI) | Cutoff value |
| --- | --- | --- | --- | --- | --- | --- |
| Sex | | | | | | |
| Female | 0.852  (0.840-0.863) | 0.814  (0.796-0.833) | 0.731  (0.712-0.750) | 0.726  (0.707-0.746) | 0.818  (0.800-0.836) | 49.310 |
| Male | 0.867  (0.855-0.878) | 0.840  (0.824-0.856) | 0.719  (0.698-0.741) | 0.783  (0.766-0.801) | 0.788  (0.767-0.809) | 53.148 |
| Race |  |  |  |  |  |  |
| Black | 0.847  (0.830-0.864) | 0.806  (0.779-0.833) | 0.726  (0.698-0.753) | 0.703  (0.674-0.732) | 0.822  (0.798-0.847) | 60.930 |
| White | 0.884  (0.872-0.897) | 0.859  (0.841-0.878) | 0.735  (0.711-0.760) | 0.781  (0.760-0.802) | 0.826  (0.804-0.849) | 49.382 |
| Hispanic | 0.823  (0.794-0.852) | 0.839  (0.803-0.874) | 0.644  (0.594-0.693) | 0.727  (0.686-0.767) | 0.779  (0.732-0.826) | 49.711 |
| Asian | 0.870  (0.847-0.893) | 0.854  (0.819-0.890) | 0.737  (0.699-0.775) | 0.705  (0.663-0.747) | 0.873  (0.842-0.904) | 35.408 |
| Mexican | 0.857  (0.831-0.883) | 0.844  (0.815-0.873) | 0.710  (0.661-0.759) | 0.840  (0.810-0.869) | 0.716  (0.668-0.765) | 50.685 |
| Other | 0.852  (0.814-0.891) | 0.709  (0.643-0.776) | 0.850  (0.798-0.902) | 0.825  (0.765-0.885) | 0.746  (0.687-0.806) | 70.746 |
| Age | | | | | | |
| <60 | 0.878  (0.869-0.887) | 0.868  (0.854-0.882) | 0.822  (0.705-0.739) | 0.735  (0.719-0.752) | 0.860  (0.845-0.874) | 46.262 |
| ≥60 | 0.814  (0.797-0.831) | 0.830  (0.810-0.849) | 0.644  (0.615-0.673) | 0.761  (0.740-0.782) | 0.734  (0.706-0.763) | 49.413 |
| Overweight | | | | | | |
| No | 0.859  (0.836-0.883) | 0.829  (0.777-0.881) | 0.777  (0.757-0.796) | 0.296  (0.258-0.334) | 0.976  (0.968-0.984) | 21.398 |
| Yes | 0.774  (0.761-0.787) | 0.684  (0.669-0.699) | 0.726  (0.706-0.746) | 0.824  (0.810-0.838) | 0.550  (0.531-0.570) | 71.768 |
| Hypertension | | | | | | |
| No | 0.873  (0.864-0.883) | 0.871  (0.857-0.886) | 0.722  (0.705-0.739) | 0.701  (0.683-0.719) | 0.883  (0.869-0.896) | 43.098 |
| Yes | 0.808  (0.790-0.825) | 0.709  (0.688-0.731) | 0.758  (0.731-0.786) | 0.843  (0.824-0.862) | 0.588  (0.560-0.615) | 71.768 |
| Diabetes | | | | | | |
| No | 0.860  (0.851-0.869) | 0.874  (0.861-0.886) | 0.683  (0.667-0.699) | 0.686  (0.671-0.702) | 0.872  (0.859-0.885) | 42.497 |
| Yes | 0.776  (0.746-0.805) | 0.737  (0.711-0.763) | 0.677  (0.626-0.728) | 0.886  (0.866-0.907) | 0.430  (0.387-0.473) | 71.549 |

Note: Black means non-Hispanic Black, White means non-Hispanic White, Hispanic means other Hispanic, Asian means non-Hispanic Asian, Mexican means Mexican American, and Other means other races.

**Supplement Table 11** Subgroup analysis of LAP for predicting MAFLD risk in the NHANES cohort

| Subgroup | AUC  (95% CI) | SEN  (95% CI) | SPE  (95% CI) | PPV  (95% CI) | NPV  (95% CI) | Cutoff value |
| --- | --- | --- | --- | --- | --- | --- |
| Sex | | | | | | |
| Female | 0.836  (0.824-0.849) | 0.795  (0.777-0.814) | 0.728  (0.708-0.747) | 0.719  (0.699-0.739) | 0.802  (0.784-0.821) | 46.603 |
| Male | 0.835  (0.822-0.848) | 0.830  (0.814-0.847) | 0.690  (0.668-0.713) | 0.764  (0.746-0.782) | 0.771  (0.750-0.792) | 40.002 |
| Race |  |  |  |  |  |  |
| Black | 0.822  (0.803-0.840) | 0.818  (0.791-0.844) | 0.692  (0.663-0.720) | 0.681  (0.652-0.710) | 0.825  (0.799-0.850) | 37.724 |
| White | 0.850  (0.835-0.864) | 0.809  (0.788-0.830) | 0.730  (0.705-0.754) | 0.767  (0.745-0.789) | 0.776  (0.752-0.800) | 47.841 |
| Hispanic | 0.795  (0.763-0.826) | 0.778  (0.737-0.818) | 0.666  (0.617-0.714) | 0.724  (0.683-0.766) | 0.726  (0.678-0.774) | 48.380 |
| Asian | 0.844  (0.819-0.869) | 0.730  (0.685-0.775) | 0.803  (0.769-0.838) | 0.732  (0.687-0.777) | 0.802  (0.767-0.836) | 46.199 |
| Mexican | 0.814  (0.783-0.844) | 0.792  (0.759-0.825) | 0.719  (0.671-0.767) | 0.835  (0.805-0.866) | 0.657  (0.609-0.706) | 46.488 |
| Other | 0.824  (0.781-0.867) | 0.743  (0.679-0.807) | 0.778  (0.717-0.839) | 0.769  (0.706-0.832) | 0.753  (0.691-0.815) | 49.479 |
| Age | | | | | | |
| <60 | 0.852  (0.842-0.863) | 0.850  (0.835-0.864) | 0.690  (0.672-0.708) | 0.709  (0.693-0.726) | 0.838  (0.822-0.853) | 38.523 |
| ≥60 | 0.787  (0.769-0.805) | 0.788  (0.767-0.809) | 0.651  (0.623-0.680) | 0.756  (0.734-0.777) | 0.692  (0.663-0.721) | 46.427 |
| Overweight | | | | | | |
| No | 0.855  (0.829-0.881) | 0.864  (0.817-0.912) | 0.689  (0.668-0.711) | 0.240  (0.208-0.271) | 0.978  (0.970-0.986) | 25.021 |
| Yes | 0.747  (0.734-0.761) | 0.672  (0.656-0.687) | 0.701  (0.680-0.721) | 0.808  (0.794-0.822) | 0.532  (0.513-0.552) | 56.914 |
| Hypertension | | | | | | |
| No | 0.849  (0.838-0.859) | 0.838  (0.822-0.854) | 0.702  (0.685-0.719) | 0.677  (0.659-0.696) | 0.853  (0.839-0.868) | 38.544 |
| Yes | 0.778  (0.759-0.796) | 0.697  (0.676-0.719) | 0.717  (0.689-0.746) | 0.819  (0.799-0.838) | 0.564  (0.536-0.592) | 57.062 |
| Diabetes | | | | | | |
| No | 0.830  (0.820-0.840) | 0.822  (0.807-0.836) | 0.681  (0.665-0.697) | 0.672  (0.655-0.688) | 0.828  (0.814-0.842) | 39.595 |
| Yes | 0.765  (0.735-0.796) | 0.679  (0.652-0.707) | 0.735  (0.687-0.783) | 0.898  (0.877-0.918) | 0.402  (0.362-0.441) | 65.709 |

Note: Black means non-Hispanic Black, White means non-Hispanic White, Hispanic means other Hispanic, Asian means non-Hispanic Asian, Mexican means Mexican American, and Other means other races.

**Supplement Table 12** Subgroup analysis of ZJU for predicting MAFLD risk in the NHANES cohort

| Subgroup | AUC  (95% CI) | SEN  (95% CI) | SPE  (95% CI) | PPV  (95% CI) | NPV  (95% CI) | Cutoff value |
| --- | --- | --- | --- | --- | --- | --- |
| Sex | | | | | | |
| Female | 0.841  (0.829-0.853) | 0.860  (0.844-0.876) | 0.663  (0.643-0.684) | 0.691  (0.672-0.711) | 0.844  (0.826-0.862) | 38.871 |
| Male | 0.869  (0.857-0.880) | 0.784  (0.766-0.802) | 0.794  (0.774-0.813) | 0.821  (0.804-0.838) | 0.752  (0.732-0.773) | 38.548 |
| Race |  |  |  |  |  |  |
| Black | 0.827  (0.808-0.845) | 0.802  (0.775-0.829) | 0.713  (0.685-0.741) | 0.692  (0.663-0.722) | 0.817  (0.792-0.842) | 40.290 |
| White | 0.866  (0.853-0.880) | 0.809  (0.789-0.830) | 0.747  (0.723-0.772) | 0.779  (0.757-0.801) | 0.781  (0.757-0.804) | 38.527 |
| Hispanic | 0.823  (0.794-0.853) | 0.768  (0.727-0.809) | 0.743  (0.698-0.788) | 0.771  (0.731-0.812) | 0.739  (0.694-0.784) | 40.214 |
| Asian | 0.880  (0.857-0.902) | 0.857  (0.822-0.892) | 0.762  (0.725-0.799) | 0.726  (0.685-0.768) | 0.879  (0.848-0.909) | 36.173 |
| Mexican | 0.839  (0.812-0.866) | 0.824  (0.793-0.854) | 0.713  (0.664-0.762) | 0.838  (0.808-0.868) | 0.692  (0.643-0.741) | 39.241 |
| Other | 0.836  (0.795-0.878) | 0.872  (0.822-0.921) | 0.694  (0.627-0.762) | 0.739  (0.680-0.799) | 0.845  (0.786-0.903) | 38.175 |
| Age | | | | | | |
| <60 | 0.870  (0.860-0.879) | 0.857  (0.843-0.871) | 0.730  (0.713-0.747) | 0.738  (0.722-0.755) | 0.852  (0.837-0.866) | 38.522 |
| ≥60 | 0.793  (0.775-0.811) | 0.733  (0.711-0.756) | 0.710  (0.682-0.737) | 0.776  (0.753-0.798) | 0.660  (0.633-0.688) | 39.229 |
| Overweight | | | | | | |
| No | 0.823  (0.794-0.853) | 0.724  (0.661-0.786) | 0.755  (0.735-0.775) | 0.251  (0.215-0.286) | 0.960  (0.950-0.971) | 33.550 |
| Yes | 0.746  (0.732-0.759) | 0.686  (0.671-0.701) | 0.688  (0.667-0.709) | 0.805  (0.791-0.819) | 0.539  (0.519-0.559) | 41.596 |
| Hypertension | | | | | | |
| No | 0.862  (0.852-0.873) | 0.873  (0.858-0.887) | 0.703  (0.686-0.720) | 0.687  (0.669-0.705) | 0.881  (0.868-0.895) | 37.386 |
| Yes | 0.791  (0.773-0.809) | 0.758  (0.738-0.778) | 0.706  (0.677-0.735) | 0.825  (0.806-0.844) | 0.614  (0.585-0.643) | 40.469 |
| Diabetes | | | | | | |
| No | 0.842  (0.832-0.851) | 0.842  (0.828-0.856) | 0.690  (0.675-0.706) | 0.683  (0.667-0.699) | 0.846  (0.832-0.860) | 37.597 |
| Yes | 0.767  (0.737-0.798) | 0.757  (0.732-0.782) | 0.655  (0.604-0.707) | 0.882  (0.862-0.903) | 0.441  (0.397-0.485) | 42.201 |

Note: Black means non-Hispanic Black, White means non-Hispanic White, Hispanic means other Hispanic, Asian means non-Hispanic Asian, Mexican means Mexican American, and Other means other races.

**Supplement Table 13** Subgroup analysis of FSI for predicting MAFLD risk in the NHANES cohort

| Subgroup | AUC  (95% CI) | SEN  (95% CI) | SPE  (95% CI) | PPV  (95% CI) | NPV  (95% CI) | Cutoff value |
| --- | --- | --- | --- | --- | --- | --- |
| Sex | | | | | | |
| Female | 0.849  (0.837-0.861) | 0.750  (0.730-0.771) | 0.774  (0.755-0.792) | 0.744  (0.724-0.764) | 0.779  (0.761-0.798) | -1.112 |
| Male | 0.867  (0.855-0.878) | 0.767  (0.748-0.785) | 0.799  (0.779-0.818) | 0.822  (0.804-0.839) | 0.739  (0.719-0.759) | -0.940 |
| Race |  |  |  |  |  |  |
| Black | 0.843  (0.826-0.861) | 0.781  (0.753-0.810) | 0.764  (0.738-0.790) | 0.727  (0.698-0.756) | 0.813  (0.788-0.837) | -0.921 |
| White | 0.877  (0.864-0.890) | 0.817  (0.796-0.837) | 0.762  (0.738-0.786) | 0.791  (0.769-0.812) | 0.791  (0.768-0.814) | -1.263 |
| Hispanic | 0.837  (0.809-0.865) | 0.802  (0.763-0.841) | 0.718  (0.672-0.765) | 0.763  (0.723-0.803) | 0.762  (0.717-0.808) | -1.100 |
| Asian | 0.875  (0.853-0.897) | 0.836  (0.799-0.873) | 0.766  (0.729-0.803) | 0.727  (0.683-0.767) | 0.864  (0.832-0.895) | -1.686 |
| Mexican | 0.846  (0.820-0.873) | 0.738  (0.703-0.774) | 0.795  (0.751-0.838) | 0.866  (0.837-0.896) | 0.628  (0.581-0.674) | -0.996 |
| Other | 0.865  (0.828-0.902) | 0.894  (0.849-0.939) | 0.661  (0.592-0.730) | 0.724  (0.665-0.783) | 0.862  (0.805-0.920) | -1.661 |
| Age | | | | | | |
| <60 | 0.881  (0.872-0.890) | 0.837  (0.822-0.852) | 0.748  (0.731-0.764) | 0.747  (0.730-0.764) | 0.838  (0.823-0.853) | -1.515 |
| ≥60 | 0.802  (0.784-0.819) | 0.736  (0.713-0.759) | 0.714  (0.687-0.742) | 0.779  (0.757-0.801) | 0.664  (0.637-0.692) | -0.829 |
| Overweight | | | | | | |
| No | 0.881  (0.858-0.903) | 0.794  (0.738-0.850) | 0.807  (0.789-0.826) | 0.318  (0.277-0.359) | 0.972  (0.963-0.980) | -2.225 |
| Yes | 0.772  (0.759-0.785) | 0.639  (0.623-0.655) | 0.774  (0.755-0.793) | 0.841  (0.827-0.855) | 0.533  (0.514-0.552) | -0.417 |
| Hypertension | | | | | | |
| No | 0.876  (0.866-0.885) | 0.871  (0.857-0.886) | 0.710  (0.693-0.727) | 0.692  (0.674-0.710) | 0.881  (0.867-0.895) | -1.889 |
| Yes | 0.805  (0.788-0.823) | 0.765  (0.745-0.785) | 0.704  (0.675-0.773) | 0.825  (0.807-0.844) | 0.620  (0.591-0.649) | -0.420 |
| Diabetes | | | | | | |
| No | 0.853  (0.844-0.862) | 0.849  (0.835-0.862) | 0.681  (0.666-0.697) | 0.679  (0.663-0.695) | 0.850  (0.837-0.864) | -1.697 |
| Yes | 0.775  (0.746-0.804) | 0.748  (0.722-0.773) | 0.674  (0.623-0.725) | 0.887  (0.866-0.907) | 0.439  (0.395-0.482) | 0.085 |

Note: Black means non-Hispanic Black, White means non-Hispanic White, Hispanic means other Hispanic, Asian means non-Hispanic Asian, Mexican means Mexican American, and Other means other races.

**Supplement Table 14** Subgroup analysis of K-NAFLD for predicting MAFLD risk in the NHANES cohort

| Subgroup | AUC  (95% CI) | SEN  (95% CI) | SPE  (95% CI) | PPV  (95% CI) | NPV  (95% CI) | Cutoff value |
| --- | --- | --- | --- | --- | --- | --- |
| Sex | | | | | | |
| Female | 0.838  (0.826-0.851) | 0.801  (0.782-0.819) | 0.710  (0.690-0.730) | 0.708  (0.688-0.728) | 0.802  (0.784-0.821) | -1.036 |
| Male | 0.844  (0.831-0.857) | 0.744  (0.725-0.763) | 0.796  (0.776-0.815) | 0.815  (0.797-0.833) | 0.720  (0.699-0.740) | -0.994 |
| Race |  |  |  |  |  |  |
| Black | 0.814  (0.795-0.834) | 0.801  (0.774-0.828) | 0.689  (0.660-0.717) | 0.674  (0.645-0.704) | 0.811  (0.785-0.837) | -1.129 |
| White | 0.855  (0.841-0.870) | 0.809  (0.789-0.830) | 0.743  (0.718-0.767) | 0.776  (0.754-0.797) | 0.780  (0.756-0.803) | -1.179 |
| Hispanic | 0.820  (0.790-0.849) | 0.790  (0.750-0.829) | 0.713  (0.666-0.759) | 0.756  (0.716-0.797) | 0.750  (0.704-0.796) | -1.049 |
| Asian | 0.852  (0.827-0.877) | 0.796  (0.756-0.837) | 0.754  (0.717-0.792) | 0.705  (0.662-0.748) | 0.834  (0.800-0.868) | -1.715 |
| Mexican | 0.826  (0.798-0.853) | 0.748  (0.713-0.783) | 0.755  (0.709-0.802) | 0.846  (0.816-0.877) | 0.625  (0.578-0.672) | -0.962 |
| Other | 0.853  (0.814-0.892) | 0.911  (0.869-0.952) | 0.667  (0.598-0.736) | 0.731  (0.673-0.789) | 0.882  (0.828-0.937) | -1.846 |
| Age | | | | | | |
| <60 | 0.865  (0.855-0.875) | 0.829  (0.814-0.844) | 0.744  (0.727-0.760) | 0.742  (0.725-0.759) | 0.830  (0.815-0.845) | -1.581 |
| ≥60 | 0.769  (0.750-0.788) | 0.667  (0.643-0.692) | 0.754  (0.727-0.780) | 0.787  (0.764-0.810) | 0.623  (0.597-0.650) | -0.242 |
| Overweight | | | | | | |
| No | 0.835  (0.807-0.863) | 0.744  (0.683-0.804) | 0.772  (0.753-0.792) | 0.270  (0.233-0.307) | 0.964  (0.954-0.974) | -2.289 |
| Yes | 0.767  (0.754-0.780) | 0.650  (0.634-0.665) | 0.771  (0.752-0.790) | 0.842  (0.828-0.856) | 0.540  (0.521-0.559) | -0.155 |
| Hypertension | | | | | | |
| No | 0.855  (0.844-0.865) | 0.799  (0.782-0.817) | 0.749  (0.732-0.765) | 0.703  (0.685-0.722) | 0.833  (0.818-0.848) | -1.589 |
| Yes | 0.771  (0.752-0.790) | 0.722  (0.701-0.743) | 0.713  (0.684-0.742) | 0.822  (0.802-0.841) | 0.584  (0.555-0.612) | -0.191 |
| Diabetes | | | | | | |
| No | 0.834  (0.824-0.844) | 0.806  (0.791-0.821) | 0.704  (0.688-0.719) | 0.684  (0.667-0.700) | 0.821  (0.807-0.835) | -1.588 |
| Yes | 0.734  (0.702-0.766) | 0.825  (0.803-0.848) | 0.548  (0.494-0.602) | 0.862  (0.841-0.882) | 0.478  (0.428-0.529) | -0.173 |

Note: Black means non-Hispanic Black, White means non-Hispanic White, Hispanic means other Hispanic, Asian means non-Hispanic Asian, Mexican means Mexican American, and Other means other races.

**Supplement Table 15** Subgroup analysis of METS-IR for predicting MAFLD risk in the Western China cohort

| Subgroup | AUC  (95% CI) | SEN  (95% CI) | SPE  (95% CI) | PPV  (95% CI) | NPV  (95% CI) | Cutoff value |
| --- | --- | --- | --- | --- | --- | --- |
| Sex | | | | | | |
| Female | 0.942  (0.931-0.952) | 0.950  (0.926-0.975) | 0.802  (0.782-0.821) | 0.470  (0.430-0.509) | 0.989  (0.983-0.994) | 31.852 |
| Male | 0.852  (0.838-0.865) | 0.803  (0.781-0.825) | 0.735  (0.714-0.756) | 0.692  (0.668-0.715) | 0.835  (0.816-0.854) | 37.322 |
| Age | | | | | | |
| <60 | 0.900  (0.892-0.909) | 0.879  (0.861-0.896) | 0.767  (0.752-0.782) | 0.631  (0.610-0.653) | 0.933  (0.923-0.943) | 35.379 |
| ≥60 | 0.864  (0.833-0.895) | 0.831  (0.776-0.885) | 0.740  (0.691-0.788) | 0.652  (0.591-0.714) | 0.881  (0.842-0.920) | 34.809 |
| Overweight | | | | | | |
| No | 0.879  (0.862-0.896) | 0.930  (0.896-0.964) | 0.699  (0.681-0.717) | 0.206  (0.180-0.231) | 0.992  (0.988-0.996) | 31.613 |
| Yes | 0.731  (0.709-0.753) | 0.627  (0.601-0.653) | 0.724  (0.692-0.756) | 0.800  (0.775-0.824) | 0.525  (0.495-0.555) | 39.962 |
| Hypertension | | | | | | |
| No | 0.899  (0.890-0.908) | 0.866  (0.847-0.885) | 0.780  (0.766-0.795) | 0.614  (0.591-0.637) | 0.935  (0.926-0.945) | 35.251 |
| Yes | 0.847  (0.817-0.877) | 0.761  (0.714-0.807) | 0.774  (0.726-0.822) | 0.790  (0.745-0.835) | 0.743  (0.694-0.793) | 38.474 |
| Diabetes | | | | | | |
| No | 0.897  (0.888-0.905) | 0.867  (0.849-0.885) | 0.769  (0.755-0.784) | 0.623  (0.602-0.644) | 0.929  (0.920-0.939) | 35.223 |
| Yes | 0.828  (0.770-0.886) | 0.756  (0.680-0.832) | 0.766  (0.672-0.861) | 0.838  (0.769-0.906) | 0.663  (0.565-0.761) | 42.106 |

**Supplement Table 16** Subgroup analysis of TyG for predicting MAFLD risk in the Western China cohort

| Subgroup | AUC  (95% CI) | SEN  (95% CI) | SPE  (95% CI) | PPV  (95% CI) | NPV  (95% CI) | Cutoff value |
| --- | --- | --- | --- | --- | --- | --- |
| Sex | | | | | | |
| Female | 0.812  (0.786-0.838) | 0.719  (0.669-0.770) | 0.764  (0.743-0.784) | 0.360  (0.322-0.399) | 0.936  (0.923-0.950) | 8.501 |
| Male | 0.711  (0.692-0.729) | 0.699  (0.674-0.724) | 0.628  (0.605-0.651) | 0.582  (0.557-0.607) | 0.738  (0.716-0.761) | 8.694 |
| Age | | | | | | |
| <60 | 0.783  (0.769-0.798) | 0.688  (0.664-0.713) | 0.744  (0.728-0.759) | 0.549  (0.526-0.573) | 0.840  (0.826-0.854) | 8.679 |
| ≥60 | 0.699  (0.652-0.746) | 0.667  (0.598-0.735) | 0.656  (0.603-0.709) | 0.533  (0.468-0.597) | 0.770  (0.719-0.820) | 8.688 |
| Overweight | | | | | | |
| No | 0.799  (0.769-0.829) | 0.670  (0.607-0.733) | 0.800  (0.784-0.815) | 0.219  (0.187-0.250) | 0.967  (0.959-0.974) | 8.694 |
| Yes | 0.651  (0.627-0.675) | 0.688  (0.663-0.713) | 0.543  (0.507-0.578) | 0.726  (0.701-0.750) | 0.498  (0.464-0.532) | 8.678 |
| Hypertension | | | | | | |
| No | 0.778  (0.763-0.792) | 0.665  (0.638-0.691) | 0.758  (0.743-0.773) | 0.526  (0.501-0.551) | 0.849  (0.835-0.862) | 8.681 |
| Yes | 0.693  (0.651-0.734) | 0.696  (0.646-0.746) | 0.664  (0.610-0.719) | 0.698  (0.649-0.748) | 0.662  (0.608-0.716) | 8.839 |
| Diabetes | | | | | | |
| No | 0.772  (0.758-0.786) | 0.788  (0.767-0.809) | 0.623  (0.607-0.640) | 0.479  (0.459-0.499) | 0.870  (0.856-0.884) | 8.482 |
| Yes | 0.662  (0.583-0.741) | 0.707  (0.627-0.788) | 0.571  (0.461-0.682) | 0.725  (0.645-0.805) | 0.550  (0.441-0.659) | 9.351 |

**Supplement Table 17** Subgroup analysis of TyG-BMI for predicting MAFLD risk in the Western China cohort

| Subgroup | AUC  (95% CI) | SEN  (95% CI) | SPE  (95% CI) | PPV  (95% CI) | NPV  (95% CI) | Cutoff value |
| --- | --- | --- | --- | --- | --- | --- |
| Sex | | | | | | |
| Female | 0.944  (0.933-0.954) | 0.931  (0.902-0.959) | 0.829  (0.811-0.847) | 0.502  (0.460-0.543) | 0.985  (0.978-0.991) | 194.427 |
| Male | 0.859  (0.847-0.872) | 0.849  (0.830-0.869) | 0.716  (0.694-0.737) | 0.689  (0.665-0.712) | 0.865  (0.848-0.883) | 213.335 |
| Age | | | | | | |
| <60 | 0.905  (0.897-0.914) | 0.838  (0.818-0.857) | 0.816  (0.803-0.830) | 0.674  (0.652-0.697) | 0.917  (0.907-0.928) | 211.515 |
| ≥60 | 0.883  (0.855-0.911) | 0.902  (0.858-0.945) | 0.707  (0.657-0.758) | 0.645  (0.586-0.703) | 0.924  (0.891-0.958) | 204.675 |
| Overweight | | | | | | |
| No | 0.897  (0.881-0.913) | 0.944  (0.914-0.975) | 0.720  (0.702-0.737) | 0.220  (0.193-0.247) | 0.994  (0.990-0.997) | 189.405 |
| Yes | 0.734  (0.713-0.756) | 0.678  (0.653-0.703) | 0.683  (0.650-0.716) | 0.790  (0.766-0.813) | 0.547  (0.515-0.578) | 228.265 |
| Hypertension | | | | | | |
| No | 0.905  (0.896-0.914) | 0.896  (0.878-0.913) | 0.762  (0.747-0.777) | 0.603  (0.580-0.625) | 0.948  (0.939-0.956) | 203.623 |
| Yes | 0.861  (0.832-0.890) | 0.758  (0.711-0.804) | 0.818  (0.774-0.863) | 0.823  (0.780-0.866) | 0.752  (0.704-0.799) | 228.178 |
| Diabetes | | | | | | |
| No | 0.903  (0.895-0.912) | 0.905  (0.890-0.920) | 0.745  (0.730-0.760) | 0.609  (0.588-0.630) | 0.947  (0.938-0.956) | 203.100 |
| Yes | 0.841  (0.785-0.897) | 0.772  (0.698-0.846) | 0.766  (0.672-0.861) | 0.841  (0.773-0.908) | 0.678  (0.580-0.776) | 237.801 |

**Supplement Table 18** Subgroup analysis of TyG-WC for predicting MAFLD risk in the Western China cohort

| Subgroup | AUC  (95% CI) | SEN  (95% CI) | SPE  (95% CI) | PPV  (95% CI) | NPV  (95% CI) | Cutoff value |
| --- | --- | --- | --- | --- | --- | --- |
| Sex | | | | | | |
| Female | 0.913  (0.898-0.928) | 0.911  (0.879-0.943) | 0.783  (0.763-0.803) | 0.437  (0.399-0.476) | 0.979  (0.972-0.987) | 643.397 |
| Male | 0.827  (0.813-0.842) | 0.788  (0.765-0.811) | 0.694  (0.673-0.716) | 0.656  (0.632-0.680) | 0.816  (0.796-0.836) | 756.988 |
| Age | | | | | | |
| <60 | 0.877  (0.867-0.887) | 0.861  (0.843-0.880) | 0.738  (0.723-0.754) | 0.599  (0.577-0.621) | 0.921  (0.911-0.932) | 714.871 |
| ≥60 | 0.835  (0.800-0.871) | 0.776  (0.716-0.836) | 0.759  (0.711-0.806) | 0.654  (0.591-0.718) | 0.852  (0.810-0.894) | 749.176 |
| Overweight | | | | | | |
| No | 0.857  (0.837-0.878) | 0.860  (0.814-0.907) | 0.719  (0.702-0.737) | 0.204  (0.178-0.230) | 0.984  (0.978-0.990) | 671.384 |
| Yes | 0.711  (0.689-0.734) | 0.579  (0.552-0.605) | 0.744  (0.713-0.775) | 0.799  (0.774-0.824) | 0.501  (0.472-0.530) | 801.486 |
| Hypertension | | | | | | |
| No | 0.874  (0.864-0.885) | 0.846  (0.826-0.866) | 0.748  (0.733-0.763) | 0.575  (0.553-0.598) | 0.923  (0.913-0.934) | 714.871 |
| Yes | 0.824  (0.792-0.857) | 0.730  (0.682-0.778) | 0.791  (0.744-0.838) | 0.796  (0.750-0.842) | 0.724  (0.675-0.773) | 794.930 |
| Diabetes | | | | | | |
| No | 0.872  (0.862-0.882) | 0.853  (0.835-0.871) | 0.736  (0.721-0.751) | 0.587  (0.566-0.608) | 0.919  (0.909-0.930) | 714.871 |
| Yes | 0.813  (0.751-0.876) | 0.878  (0.820-0.936) | 0.610  (0.501-0.719) | 0.783  (0.714-0.851) | 0.758  (0.651-0.865) | 818.179 |

**Supplement Table 19** Subgroup analysis of TyG-WtHR for predicting MAFLD risk in the Western China cohort

| Subgroup | AUC  (95% CI) | SEN  (95% CI) | SPE  (95% CI) | PPV  (95% CI) | NPV  (95% CI) | Cutoff value |
| --- | --- | --- | --- | --- | --- | --- |
| Sex | | | | | | |
| Female | 0.906  (0.891-0.922) | 0.875  (0.837-0.912) | 0.802  (0.782-0.821) | 0.449  (0.409-0.489) | 0.972  (0.963-0.981) | 4.119 |
| Male | 0.814  (0.799-0.829) | 0.766  (0.743-0.790) | 0.701  (0.679-0.723) | 0.655  (0.630-0.679) | 0.802  (0.782-0.822) | 4.478 |
| Age | | | | | | |
| <60 | 0.872  (0.862-0.882) | 0.896  (0.879-0.912) | 0.692  (0.676-0.709) | 0.569  (0.548-0.590) | 0.936  (0.926-0.946) | 4.198 |
| ≥60 | 0.830  (0.795-0.866) | 0.607  (0.563-0.677) | 0.887  (0.852-0.923) | 0.760  (0.691-0.830) | 0.793  (0.751-0.836) | 4.861 |
| Overweight | | | | | | |
| No | 0.853  (0.831-0.875) | 0.828  (0.777-0.878) | 0.744  (0.727-0.761) | 0.213  (0.185-0.241) | 0.981  (0.975-0.987) | 4.113 |
| Yes | 0.691  (0.668-0.714) | 0.499  (0.472-0.526) | 0.786  (0.757-0.815) | 0.804  (0.777-0.831) | 0.472  (0.444-0.499) | 4.881 |
| Hypertension | | | | | | |
| No | 0.867  (0.856-0.878) | 0.887  (0.869-0.904) | 0.697  (0.681-0.713) | 0.542  (0.520-0.563) | 0.938  (0.928-0.948) | 4.198 |
| Yes | 0.817  (0.784-0.850) | 0.840  (0.801-0.880) | 0.651  (0.596-0.705) | 0.729  (0.684-0.774) | 0.785  (0.733-0.837) | 4.594 |
| Diabetes | | | | | | |
| No | 0.865  (0.855-0.875) | 0.896  (0.880-0.912) | 0.679  (0.663-0.696) | 0.551  (0.531-0.571) | 0.937  (0.927-0.947) | 4.198 |
| Yes | 0.825  (0.764-0.887) | 0.837  (0.772-0.903) | 0.701  (0.599-0.804) | 0.817  (0.750-0.885) | 0.730  (0.629-0.831) | 4.990 |

**Supplement Table 20** Subgroup analysis of HSI for predicting MAFLD risk in the Western China cohort

| Subgroup | AUC  (95% CI) | SEN  (95% CI) | SPE  (95% CI) | PPV  (95% CI) | NPV  (95% CI) | Cutoff value |
| --- | --- | --- | --- | --- | --- | --- |
| Sex | | | | | | |
| Female | 0.909  (0.894-0.924) | 0.927  (0.898-0.957) | 0.754  (0.733-0.775) | 0.411  (0.374-0.448) | 0.983  (0.975-0.990) | 31.602 |
| Male | 0.832  (0.817-0.846) | 0.793  (0.770-0.815) | 0.716  (0.695-0.738) | 0.674  (0.650-0.698) | 0.824  (0.804-0.843) | 34.075 |
| Age | | | | | | |
| <60 | 0.878  (0.867-0.888) | 0.817  (0.796-0.837) | 0.776  (0.761-0.790) | 0.623  (0.600-0.645) | 0.903  (0.892-0.915) | 33.739 |
| ≥60 | 0.859  (0.827-0.891) | 0.809  (0.752-0.866) | 0.759  (0.711-0.806) | 0.664  (0.602-0.726) | 0.871  (0.831-0.911) | 32.413 |
| Overweight | | | | | | |
| No | 0.807  (0.781-0.833) | 0.698  (0.636-0.759) | 0.772  (0.755-0.788) | 0.204  (0.175-0.233) | 0.968  (0.961-0.976) | 31.706 |
| Yes | 0.735  (0.714-0.757) | 0.527  (0.500-0.553) | 0.828  (0.801-0.855) | 0.843  (0.818-0.868) | 0.499  (0.471-0.526) | 37.467 |
| Hypertension | | | | | | |
| No | 0.872  (0.861-0.882) | 0.879  (0.861-0.898) | 0.703  (0.686-0.719) | 0.544  (0.522-0.566) | 0.935  (0.925-0.945) | 32.387 |
| Yes | 0.860  (0.832-0.888) | 0.724  (0.675-0.772) | 0.836  (0.793-0.878) | 0.831  (0.787-0.875) | 0.731  (0.683-0.778) | 35.213 |
| Diabetes | | | | | | |
| No | 0.872  (0.862-0.883) | 0.834  (0.815-0.853) | 0.745  (0.730-0.760) | 0.590  (0.568-0.611) | 0.911  (0.900-0.922) | 33.032 |
| Yes | 0.816  (0.759-0.873) | 0.569  (0.482-0.657) | 0.922  (0.862-0.982) | 0.921  (0.860-0.982) | 0.573  (0.486-0.660) | 38.928 |

**Supplement Table 21** Subgroup analysis of VAI for predicting MAFLD risk in the Western China cohort

| Subgroup | AUC  (95% CI) | SEN  (95% CI) | SPE  (95% CI) | PPV  (95% CI) | NPV  (95% CI) | Cutoff value |
| --- | --- | --- | --- | --- | --- | --- |
| Sex | | | | | | |
| Female | 0.824  (0.800-0.849) | 0.772  (0.725-0.819) | 0.743  (0.722-0.764) | 0.357  (0.321-0.394) | 0.946  (0.934-0.959) | 1.405 |
| Male | 0.721  (0.702-0.739) | 0.649  (0.622-0.675) | 0.690  (0.668-0.712) | 0.607  (0.581-0.633) | 0.726  (0.704-0.748) | 1.782 |
| Age | | | | | | |
| <60 | 0.781  (0.767-0.796) | 0.768  (0.746-0.791) | 0.658  (0.641-0.675) | 0.505  (0.484-0.527) | 0.862  (0.848-0.876) | 1.423 |
| ≥60 | 0.701  (0.654-0.747) | 0.596  (0.525-0.667) | 0.730  (0.681-0.779) | 0.565  (0.495-0.635) | 0.754  (0.706-0.803) | 1.636 |
| Overweight | | | | | | |
| No | 0.785  (0.754-0.816) | 0.763  (0.706-0.820) | 0.685  (0.667-0.703) | 0.169  (0.145-0.192) | 0.972  (0.964-0.979) | 1.347 |
| Yes | 0.662  (0.638-0.686) | 0.622  (0.596-0.648) | 0.635  (0.600-0.669) | 0.750  (0.724-0.775) | 0.489  (0.458-0.520) | 1.852 |
| Hypertension | | | | | | |
| No | 0.776  (0.761-0.791) | 0.742  (0.718-0.767) | 0.672  (0.655-0.688) | 0.477  (0.455-0.500) | 0.866  (0.852-0.880) | 1.421 |
| Yes | 0.698  (0.657-0.739) | 0.721  (0.672-0.770) | 0.599  (0.543-0.656) | 0.668  (0.618-0.717) | 0.658  (0.601-0.715) | 1.741 |
| Diabetes | | | | | | |
| No | 0.770  (0.756-0.784) | 0.749  (0.726-0.771) | 0.658  (0.642-0.674) | 0.490  (0.469-0.511) | 0.856  (0.843-0.870) | 1.416 |
| Yes | 0.706  (0.630-0.782) | 0.797  (0.726-0.868) | 0.571  (0.461-0.682) | 0.748  (0.674-0.822) | 0.638  (0.524-0.751) | 1.872 |

**Supplement Table 22** Subgroup analysis of FLI for predicting MAFLD risk in the Western China cohort

| Subgroup | AUC  (95% CI) | SEN  (95% CI) | SPE  (95% CI) | PPV  (95% CI) | NPV  (95% CI) | Cutoff value |
| --- | --- | --- | --- | --- | --- | --- |
| Sex | | | | | | |
| Female | 0.925  (0.911-0.938) | 0.822  (0.779-0.865) | 0.890  (0.874-0.905) | 0.579  (0.532-0.626) | 0.964  (0.955-0.974) | 17.485 |
| Male | 0.832  (0.817-0.846) | 0.849  (0.830-0.869) | 0.651  (0.628-0.673) | 0.643  (0.620-0.666) | 0.854  (0.835-0.873) | 31.653 |
| Age | | | | | | |
| <60 | 0.882  (0.872-0.892) | 0.872  (0.854-0.890) | 0.738  (0.722-0.754) | 0.602  (0.580-0.623) | 0.927  (0.917-0.938) | 24.536 |
| ≥60 | 0.847  (0.814-0.880) | 0.945  (0.912-0.978) | 0.592  (0.537-0.646) | 0.577  (0.521-0.633) | 0.948  (0.917-0.980) | 17.331 |
| Overweight | | | | | | |
| No | 0.866  (0.847-0.885) | 0.847  (0.798-0.895) | 0.782  (0.766-0.798) | 0.246  (0.215-0.277) | 0.984  (0.978-0.989) | 17.343 |
| Yes | 0.720  (0.698-0.742) | 0.609  (0.583-0.635) | 0.715  (0.683-0.747) | 0.790  (0.765-0.814) | 0.510  (0.480-0.540) | 49.158 |
| Hypertension | | | | | | |
| No | 0.880  (0.870-0.890) | 0.852  (0.832-0.871) | 0.756  (0.741-0.771) | 0.585  (0.562-0.608) | 0.927  (0.916-0.937) | 24.576 |
| Yes | 0.830  (0.798-0.862) | 0.736  (0.688-0.784) | 0.781  (0.733-0.828) | 0.789  (0.744-0.835) | 0.726  (0.677-0.775) | 47.072 |
| Diabetes | | | | | | |
| No | 0.878  (0.868-0.887) | 0.854  (0.836-0.873) | 0.741  (0.726-0.756) | 0.592  (0.571-0.613) | 0.921  (0.910-0.931) | 24.536 |
| Yes | 0.827  (0.767-0.887) | 0.862  (0.801-0.923) | 0.649  (0.543-0.756) | 0.797  (0.729-0.865) | 0.746  (0.642-0.850) | 39.298 |

**Supplement Table 23** Subgroup analysis of LAP for predicting MAFLD risk in the Western China cohort

| Subgroup | AUC  (95% CI) | SEN  (95% CI) | SPE  (95% CI) | PPV  (95% CI) | NPV  (95% CI) | Cutoff value |
| --- | --- | --- | --- | --- | --- | --- |
| Sex | | | | | | |
| Female | 0.896  (0.878-0.913) | 0.871  (0.834-0.909) | 0.769  (0.749-0.790) | 0.411  (0.373-0.449) | 0.970  (0.961-0.979) | 20.940 |
| Male | 0.797  (0.781-0.813) | 0.800  (0.778-0.822) | 0.655  (0.632-0.677) | 0.631  (0.608-0.655) | 0.816  (0.795-0.836) | 30.700 |
| Age | | | | | | |
| <60 | 0.859  (0.848-0.870) | 0.816  (0.795-0.836) | 0.746  (0.730-0.761) | 0.593  (0.571-0.615) | 0.899  (0.887-0.911) | 28.430 |
| ≥60 | 0.800  (0.762-0.838) | 0.743  (0.680-0.806) | 0.727  (0.677-0.776) | 0.615  (0.551-0.680) | 0.828  (0.783-0.873) | 32.535 |
| Overweight | | | | | | |
| No | 0.850  (0.828-0.872) | 0.819  (0.767-0.870) | 0.728  (0.711-0.745) | 0.201  (0.175-0.228) | 0.980  (0.973-0.986) | 21.730 |
| Yes | 0.697  (0.674-0.720) | 0.565  (0.538-0.591) | 0.733  (0.702-0.765) | 0.788  (0.762-0.814) | 0.489  (0.460-0.518) | 45.675 |
| Hypertension | | | | | | |
| No | 0.856  (0.844-0.867) | 0.794  (0.772-0.817) | 0.759  (0.744-0.774) | 0.571  (0.548-0.595) | 0.901  (0.890-0.913) | 28.430 |
| Yes | 0.793  (0.758-0.828) | 0.650  (0.599-0.702) | 0.798  (0.752-0.844) | 0.782  (0.733-0.831) | 0.671  (0.622-0.721) | 47.830 |
| Diabetes | | | | | | |
| No | 0.853  (0.842-0.863) | 0.800  (0.779-0.821) | 0.746  (0.731-0.761) | 0.580  (0.558-0.602) | 0.894  (0.883-0.906) | 28.720 |
| Yes | 0.782  (0.713-0.851) | 0.764  (0.689-0.839) | 0.701  (0.599-0.804) | 0.803  (0.731-0.875) | 0.651  (0.548-0.753) | 48.535 |

**Supplement Table 24** Subgroup analysis of ZJU for predicting MAFLD risk in the Western China cohort

| Subgroup | AUC  (95% CI) | SEN  (95% CI) | SPE  (95% CI) | PPV  (95% CI) | NPV  (95% CI) | Cutoff value |
| --- | --- | --- | --- | --- | --- | --- |
| Sex | | | | | | |
| Female | 0.942  (0.932-0.952) | 0.904  (0.871-0.937) | 0.851  (0.834-0.868) | 0.529  (0.486-0.572) | 0.980  (0.972-0.987) | 34.433 |
| Male | 0.862  (0.849-0.874) | 0.848  (0.828-0.868) | 0.703  (0.681-0.725) | 0.678  (0.655-0.702) | 0.862  (0.844-0.880) | 34.728 |
| Age | | | | | | |
| <60 | 0.902  (0.894-0.911) | 0.856  (0.838-0.875) | 0.790  (0.776-0.805) | 0.649  (0.627-0.671) | 0.924  (0.913-0.934) | 34.728 |
| ≥60 | 0.873  (0.843-0.903) | 0.809  (0.752-0.866) | 0.785  (0.739-0.830) | 0.688  (0.626-0.750) | 0.875  (0.836-0.913) | 35.039 |
| Overweight | | | | | | |
| No | 0.878  (0.860-0.895) | 0.912  (0.874-0.950) | 0.701  (0.683-0.719) | 0.203  (0.178-0.229) | 0.990  (0.985-0.994) | 32.297 |
| Yes | 0.748  (0.727-0.769) | 0.675  (0.650-0.700) | 0.702  (0.669-0.734) | 0.799  (0.776-0.822) | 0.552  (0.520-0.583) | 37.109 |
| Hypertension | | | | | | |
| No | 0.901  (0.892-0.910) | 0.870  (0.852-0.889) | 0.773  (0.758-0.788) | 0.607  (0.584-0.630) | 0.937  (0.927-0.946) | 34.310 |
| Yes | 0.859  (0.830-0.888) | 0.828  (0.787-0.869) | 0.740  (0.689-0.790) | 0.780  (0.737-0.824) | 0.794  (0.746-0.842) | 36.164 |
| Diabetes | | | | | | |
| No | 0.902  (0.894-0.911) | 0.854  (0.835-0.872) | 0.786  (0.772-0.800) | 0.637  (0.616-0.659) | 0.924  (0.915-0.934) | 34.549 |
| Yes | 0.790  (0.723-0.857) | 0.837  (0.772-0.903) | 0.623  (0.515-0.732) | 0.780  (0.710-0.851) | 0.706  (0.598-0.814) | 39.523 |

**Supplement Table 25** Subgroup analysis of HSI for predicting MAFLD risk in the Western China cohort

| Subgroup | AUC  (95% CI) | SEN  (95% CI) | SPE  (95% CI) | PPV  (95% CI) | NPV  (95% CI) | Cutoff value |
| --- | --- | --- | --- | --- | --- | --- |
| Sex | | | | | | |
| Female | 0.921  (0.908-0.934) | 0.875  (0.837-0.912) | 0.827  (0.808-0.845) | 0.483  (0.441-0.524) | 0.973  (0.964-0.981) | -2.633 |
| Male | 0.817  (0.802-0.832) | 0.692  (0.666-0.717) | 0.779  (0.759-0.799) | 0.698  (0.673-0.724) | 0.774  (0.754-0.794) | -1.463 |
| Age | | | | | | |
| <60 | 0.878  (0.868-0.887) | 0.900  (0.884-0.916) | 0.691  (0.675-0.708) | 0.570  (0.549-0.590) | 0.938  (0.928-0.948) | -2.408 |
| ≥60 | 0.822  (0.786-0.857) | 0.787  (0.728-0.846) | 0.688  (0.637-0.740) | 0.598  (0.536-0.659) | 0.846  (0.801-0.890) | -1.989 |
| Overweight | | | | | | |
| No | 0.864  (0.844-0.883) | 0.842  (0.793-0.891) | 0.751  (0.734-0.768) | 0.221  (0.192-0.249) | 0.983  (0.977-0.988) | -2.591 |
| Yes | 0.721  (0.699-0.743) | 0.622  (0.596-0.648) | 0.728  (0.696-0.760) | 0.801  (0.776-0.825) | 0.523  (0.493-0.553) | -1.257 |
| Hypertension | | | | | | |
| No | 0.875  (0.865-0.886) | 0.903  (0.886-0.920) | 0.699  (0.683-0.716) | 0.548  (0.526-0.570) | 0.947  (0.938-0.956) | -2.489 |
| Yes | 0.834  (0.802-0.866) | 0.840  (0.801-0.880) | 0.695  (0.642-0.748) | 0.755  (0.711-0.799) | 0.796  (0.747-0.846) | -1.320 |
| Diabetes | | | | | | |
| No | 0.873  (0.863-0.883) | 0.896  (0.880-0.912) | 0.691  (0.675-0.707) | 0.560  (0.540-0.581) | 0.938  (0.928-0.947) | -2.408 |
| Yes | 0.810  (0.745-0.874) | 0.789  (0.716-0.861) | 0.766  (0.672-0.861) | 0.843  (0.777-0.910) | 0.694  (0.596-0.792) | -0.499 |

**Supplement Table 26** Subgroup analysis of K-NAFLD for predicting MAFLD risk in the Western China cohort

| Subgroup | AUC  (95% CI) | SEN  (95% CI) | SPE  (95% CI) | PPV  (95% CI) | NPV  (95% CI) | Cutoff value |
| --- | --- | --- | --- | --- | --- | --- |
| Sex | | | | | | |
| Female | 0.876  (0.858-0.894) | 0.855  (0.815-0.894) | 0.775  (0.755-0.795) | 0.412  (0.374-0.451) | 0.967  (0.957-0.976) | -2.734 |
| Male | 0.796  (0.780-0.812) | 0.757  (0.734-0.781) | 0.692  (0.670-0.714) | 0.645  (0.621-0.670) | 0.794  (0.774-0.815) | -2.240 |
| Age | | | | | | |
| <60 | 0.843  (0.832-0.855) | 0.809  (0.788-0.829) | 0.733  (0.717-0.749) | 0.579  (0.557-0.601) | 0.894  (0.882-0.906) | -2.588 |
| ≥60 | 0.758  (0.716-0.800) | 0.639  (0.570-0.709) | 0.740  (0.691-0.788) | 0.591  (0.522-0.659) | 0.777  (0.730-0.824) | -1.665 |
| Overweight | | | | | | |
| No | 0.816  (0.789-0.842) | 0.809  (0.757-0.862) | 0.706  (0.688-0.724) | 0.187  (0.162-0.212) | 0.978  (0.971-0.985) | -3.202 |
| Yes | 0.713  (0.691-0.736) | 0.616  (0.590-0.642) | 0.712  (0.680-0.744) | 0.790  (0.765-0.815) | 0.514  (0.484-0.544) | -1.217 |
| Hypertension | | | | | | |
| No | 0.834  (0.822-0.847) | 0.768  (0.744-0.791) | 0.758  (0.743-0.773) | 0.561  (0.538-0.585) | 0.890  (0.878-0.902) | -2.589 |
| Yes | 0.808  (0.774-0.842) | 0.620  (0.567-0.672) | 0.860  (0.820-0.899) | 0.831  (0.784-0.878) | 0.669  (0.622-0.717) | 0.191 |
| Diabetes | | | | | | |
| No | 0.835  (0.823-0.847) | 0.794  (0.773-0.815) | 0.729  (0.714-0.745) | 0.563  (0.541-0.585) | 0.889  (0.877-0.901) | -2.589 |
| Yes | 0.748  (0.678-0.819) | 0.683  (0.601-0.765) | 0.714  (0.613-0.815) | 0.792  (0.715-0.870) | 0.585  (0.486-0.685) | 1.084 |
